# Epigenetic priming of immune/inflammatory pathways activation and abnormal activity of cell cycle pathway in a perinatal model of white matter injury

**DOI:** 10.1038/s41419-022-05483-4

**Published:** 2022-12-13

**Authors:** Anne-Laure Schang, Juliette Van Steenwinckel, Zoi S. Ioannidou, Julia Lipecki, Charlotte Rich-Griffin, Kate Woolley-Allen, Nigel Dyer, Tifenn Le Charpentier, Patrick Schäfer, Bobbi Fleiss, Sascha Ott, Délara Sabéran-Djoneidi, Valérie Mezger, Pierre Gressens

**Affiliations:** 1 Université Paris Cité, Epigenetics and Cell Fate, CNRS, F-75013 Paris, France; 2 Université Paris Cité, NeuroDiderot, Inserm, F-75019 Paris, France; 3 School of Life Sciences, University of Warwick, Coventry, CV4 7AL UK; 4 Bioinformatics Research Technology Platform, Warwick University, Coventry, CV4 7AL UK; 5 Warwick Medical School, University of Warwick, Coventry, CV4 7AL UK; 6 Present Address: Inserm, UMR1153, Epidemiology and Biostatistics Sorbonne Paris Cité Center (CRESS) HERA team. Université Paris Cité, Faculté de Santé, Faculté de Pharmacie de Paris, 4 avenue de l’Observatoire, 75006 Paris, France; 7 Present Address: School of Health and Biomedical Sciences, RMIT University, Bundoora, VIC Australia

**Keywords:** Epigenetic memory, Molecular neuroscience, Developmental neurogenesis

## Abstract

Prenatal inflammatory insults accompany prematurity and provoke diffuse white matter injury (DWMI), which is associated with increased risk of neurodevelopmental pathologies, including autism spectrum disorders. DWMI results from maturation arrest of oligodendrocyte precursor cells (OPCs), a process that is poorly understood. Here, by using a validated mouse model of OPC maturation blockade, we provide the genome-wide ID card of the effects of neuroinflammation on OPCs that reveals the architecture of global cell fate issues underlining their maturation blockade. First, we find that, in OPCs, neuroinflammation takes advantage of a primed epigenomic landscape and induces abnormal overexpression of genes of the immune/inflammatory pathways: these genes strikingly exhibit accessible chromatin conformation in uninflamed OPCs, which correlates with their developmental, stage-dependent expression, along their normal maturation trajectory, as well as their abnormal upregulation upon neuroinflammation. Consistently, we observe the positioning on DNA of key transcription factors of the immune/inflammatory pathways (IRFs, NFkB), in both unstressed and inflamed OPCs. Second, we show that, in addition to the general perturbation of the myelination program, neuroinflammation counteracts the physiological downregulation of the cell cycle pathway in maturing OPCs. Neuroinflammation therefore perturbs cell identity in maturing OPCs, in a global manner. Moreover, based on our unraveling of the activity of genes of the immune/inflammatory pathways in prenatal uninflamed OPCs, the mere suppression of these proinflammatory mediators, as currently proposed in the field, may not be considered as a valid neurotherapeutic strategy.

## Introduction

Premature birth, i.e. birth before 37 of 40 completed weeks, occurs in 8–13% of all births worldwide and is the commonest cause of death and disability in children under 5 years of age [1]. Life-long morbidity is predominantly due to neurological damage, which includes an array of effects, collectively called “encephalopathy of prematurity” [2]. Almost 10% of infants born before 33 weeks develop cerebral palsy and approximately 35% have persistent cognitive and neuropsychiatric deficits, including autism spectrum disorders and attention deficit/hyperactivity disorder [3]. Although the most severe problems stem from extreme prematurity, even slight reductions in gestational length have significant adverse effects. One of the hallmarks of encephalopathy of prematurity is diffuse white matter injury (DWMI), which is considered a key target for neuroprotection and the prevention of long-lasting handicap. DWMI is due to oligodendrocyte maturation arrest, leading to hypomyelination and ultimately to defects in gray matter connectivity [1, 4, 5]. In that context, neuroinflammation is a leading cause of encephalopathy of prematurity, serving as a central mediator of oligodendrocyte maturation defects and hypomyelination [6, 7]. However, the delicately choreographed programs that control OPC maturation and their perturbation by neuroinflammation are not understood and remain to be enlightened.

To do so, we took advantage of a mouse model of DWMI that we previously validated and which recapitulates the induction by neuroinflammation of arrest in oligodendrocyte maturation, long-term hypomyelination, and cognitive deficits, as seen clinically [8–11]. Using purified populations of premyelinating OPCs and immature oligodendrocytes isolated from this animal model, based on their expressing the O4 cell surface marker, we have genome-widely and molecularly characterized the global cell fate issues that underlie OPC maturation blockade, in response to neuroinflammation. We showed that the transcriptomes of these O4+ cell populations are majorly disturbed by neuroinflammation. First, our analyses strikingly revealed that, in O4+ cells at postnatal day 5 (P5), the most significantly perturbed pathway corresponded to the immune system and inflammatory response and that these major transcriptional perturbations occurred without marked epigenomic alterations. Indeed, we found that neuroinflammation operated in an open epigenetic landscape on numerous genes of the innate immune/inflammatory pathway, which were already transcriptionally active in uninflamed P5 O4+ cells, and counteracted their programmed downregulation in P10 O4+ cell populations, by inducing abnormal and sustained overexpression. Consistently, we observe the positioning on DNA of key transcription factors of the immune/inflammatory pathways (IRFs, NFkB, etc.), in both unstressed and inflamed P5 O4+ cells. Importantly, we recapitulate our findings in an oligodendroglial cell line. Altogether, this indicates that neuroinflammation acts by taking advantage of a primed chromatin landscape in P5 O4+ cells to globally rewire, in a deleterious manner, the transcriptional outcome of gene of the immune/inflammatory pathway. Second, in P10 O4+ cell populations, the genome-wide impact of neuroinflammation was not only signed by the general alteration of the myelination program, as expected in our model, but also by the global induction of genes belonging to cell cycle pathway. We thus provide, through these genome-wide unbiased analyses, a global profiling of the impact of neuroinflammation, which acts on three major pathways—immune/inflammatory, cell cycle, and myelination—leading to an “in-between”-frozen cell fate that reflects and likely underlies the OPC maturation blockade.

Finally, the corollary of the unexpected and developmentally regulated expression of numerous inflammatory genes by P5 O4+ cells during their normal stage-dependent maturation has potentially important implications for therapeutic strategies: the blanket, pharmacological repression of the immune/inflammatory pathway in newborns is likely not a valid strategy.

## Results

### Validation of the maturation blockade in O4+-purified OPCs under neuroinflammation conditions

**Fig. 1:**
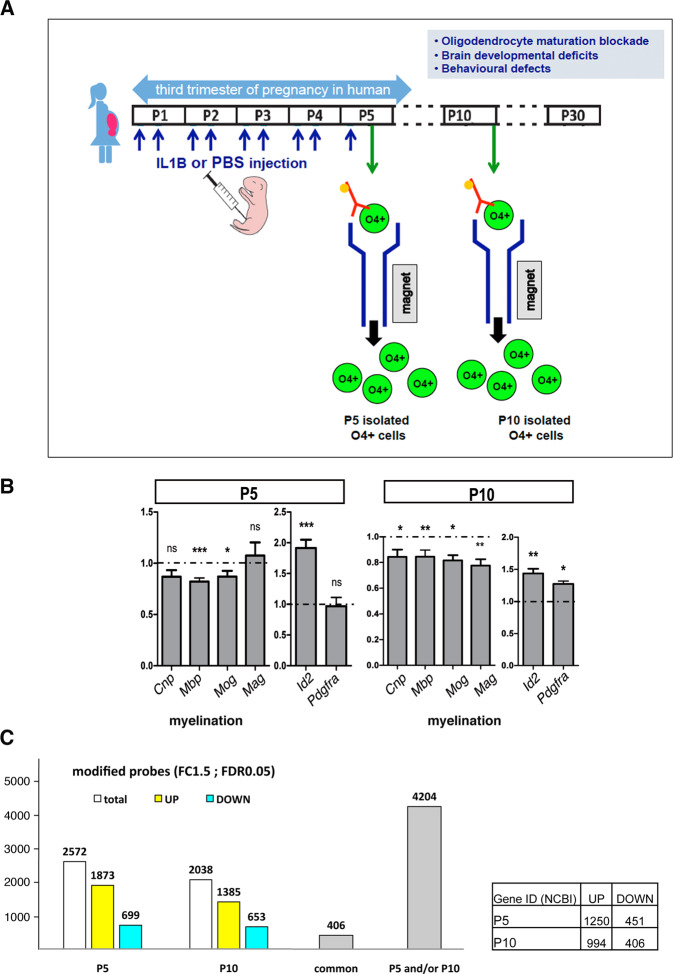
Experimental strategy and major transcriptomic impact of neuroinflammation on the immune/inflammatory pathway in O4+ cells. **A** We used a mouse model of encephalopathy of prematurity that we previously validated and in which we mimic the systemic and neuroinflammatory insults as undergone by human infants from approximately (23–32)-week gestational age equivalent. Neuroinflammation is induced *via* i.p. IL1B from postnatal days 1–5, and this leads to OPC maturation blockade, defective myelination, and behavioral anomalies as seen clinically [8]. O4+ cell populations (green circles) were respectively isolated from P5 and P10 pup cortices by MACS (see “Materials and methods”). **B** Quality-control validation of OPC maturation arrest in the model of perinatal neuroinflammation. RT-qPCR analysis of the expression of myelination and progenitor markers in O4+ cells showing that neuroinflammation, induced by IL1B peritoneal injections in pups, results in defects in the myelination gene expression program and inappropriate elevation of progenitor markers at P5 and P10. Myelin markers: *Mbp*, Myelin binding protein; *Mog*, Myelin oligodendrocyte glycoprotein; *Mag* Myelin-associated glycoprotein. *Plp1* proteolipid protein 1, a transmembrane, predominant component of myelin; *Cnp*, 2′,3′-Cyclic Nucleotide 3′ Phosphodiesterase, abundant protein in myelin in the central nervous system. Progenitor (OPC) markers: *Id2*, Inhibitor of differentiation 2; *Pdgfra*, Platelet Derived Growth Factor Receptor Alpha. Number of independent experiments at P5: *n* = 7 for *Cnp*, *Mag*, and *Pdgfra*; and *n* = 15 for *Mbp*, *Mog*, and *Id2*; at P10: *n* = 14 for *Mag* and *Mog*; and *n* = 19 for *Cnp*, *Pdgfra*; and *Mbp*, and *Id2*. ns not statistically significant; *p < 0.05; ***p < 0.001. **C** (Left) Graphical representation of the total number of probes dysregulated upon neuroinflammation induced by IL1B injections in O4+ cells at P5 and P10 (white; 2572 probes at P5 and 2038 probes at P10) including upregulated (yellow; 1873 probes at P5 and 1385 probes at P10) and downregulated (blue; 699 probes at P5 and 653 probes at P10) probes; gray bars: number of probes dysregulated at P5 and P10 (common), and dysregulated at least at one stage (P5 and/or P10). (Right) Number of upregulated (UP) and downregulated genes (DOWN), at P5 or P10.

In our in vivo model of neuroinflammation, the common exposure of preterm-born infants to systemic and central inflammation (neuroinflammation) is mimicked by intraperitoneal (i.p.) administration of interleukin 1B (IL1B) from postnatal days 1–5 (P1–P5; (Fig. 1A)). Because in clinical conditions and diverse models of DWMI, males are more severely affected than females [7], many studies on oligodendrocyte precursor cells (OPCs) have been performed in male animals, and so has the present study. The developmental window (P1–P5) is equivalent to the high-risk window for encephalopathy of prematurity in infants, (23–32)-week gestational age. In our previous studies, we have demonstrated that oligodendrocyte maturation arrest is a hallmark of the neuropathy caused by neuroinflammation that is triggered by intraperitoneal IL1B administration [8–11].

Using magnetic-activated cell sorting (MACS), we isolated, from male cortices, in each condition, the premyelinating cell populations of the oligodendrocyte lineage at P5 and P10 that express the O4 cell surface antigen (Fig. 1A). These O4+ cell population contain late OPCs and very immature OLs (immOLs), with different proportions between P5 and P10. They were recently shown to be very sensitive to neuroinflammatory stimulus especially as compared with PDGFRα+/O4− cells [2, 12]. Of note, O4+ cells were directly isolated from pup cortices, and immediately and directly processed without culturing. We assessed, by RT-qPCR experiments, that this protocol of induction of neuroinflammation reproducibly and significantly increased the expression of *Id2*, a transcriptional inhibitor of oligodendrocyte differentiation, whereas it reduced that of myelination-associated genes (*Cnp*, *Mbp*, *Mog*, and *Mag*), as expected from our previous studies (Fig. 1B; [8]).

### Major transcriptomic impact of neuroinflammation on the immune/inflammatory pathway in O4+ OPCs

We first investigated the impact of neuroinflammation on the transcriptome of O4+ cells, using microarray analysis. We compared six independent samples of O4+ cells at P5 and O4+ cells at P10 from IL1B-exposed mice to six independent samples from PBS-treated (control) mice at each stage. We found that neuroinflammation mainly triggered the upregulation of gene expression in O4+ cells: 1250 genes (unique NCBI Gene ID) were upregulated versus 451 downregulated at P5, which corresponded to 1873 and 699 probes, respectively (FC ±1.5; FDR < 0.05; Fig. 1C; NCBI Gene Expression Omnibus; accession no: GEO GSE197563 and Table S1). Similarly, at P10, 994 genes were upregulated and 406 downregulated upon neuroinflammation, corresponding to 1,385 and 653 probes (FC ±1.5; FDR < 0.05; Fig. 1C; GEO GSE197563 and Table S1).

**Fig. 2:**
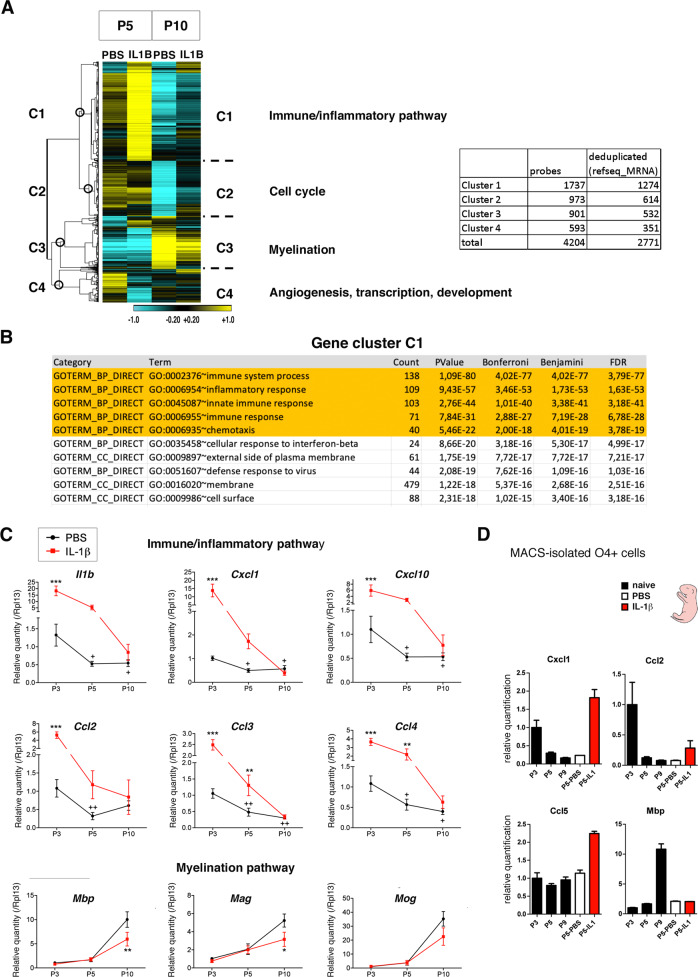
The overall physiological and dynamic expression of genes of the immune system and inflammatory response is perturbed by neuroinflammation (See also Table S1). Microarray analysis comparing gene expression in isolated O4+ cells from six independent control (PBS) and six independent neuroinflammation-exposed cortices (IL1B; see Fig. 1). **A** Major transcriptomic activation of the immune/inflammatory pathways in O4+ cells (cluster C1) by neuroinflammation, concomitant to alteration of cell cycle (cluster C2), myelination gene (cluster C3), and angiogenesis (Cluster C4) gene expression programs. (Left) Heat map of genes showing dynamic changes of expression between P5 and P10 in response to neuroinflammation. A fold-change (FC) threshold of ±1.5 was chosen, with FDR < 0.05. The heat map is centered around the median of the 6 values, with the color scale: log2 [−1.0; +1.0]. Transcriptomic analysis reveals 4 groups of genes with distinct expression profiles (clusters C1 to C4). Hierarchical clustering, rows, one minus Pearson correlation, linkage method: average. (Right) Total numbers of probes and corresponding genes belonging to clusters C1 to C4. **B** The top5 GO-terms of upregulated genes in P5 O4+ cells correspond to immune system and inflammatory response pathways. **C** Constitutive expression of genes encoding cytokines and chemokines at early stages of normal (unstressed) O4+ OPC maturation. RT-qPCR analyses in P3, P5, and P10 isolated O4+ cells. *N* = 5–6 independent experiments. Two-way ANOVA followed by Bonferroni - Post Hoc Test was performed: * and +*p* < 0.05; ** and, ++*p* < 0.01; ***p < 0.001. *, **, or ***, correspond to comparison between PBS and IL1 conditions for a given postnatal stage; + (or++), correspond to comparison between P5 (or P10) to P3. Myelination genes are plotted as a control of the effect of neuroinflammation on O4+ cells at P10. Note that in this set of experiments the downregulation of myelination genes by neuroinflammation was not observed at P5, a stage at which these genes only start to be expressed. **D** O4+ cells from naïve pups also constitutively express cytokine and chemokine genes. RT-qPCR experiments comparing O4+ cells from naïve (no injection), PBS-injected and IL1B-injected pups, at P3 to P9. PBS injection does not per se induce the expression of cytokines and chemokine genes, since the equivalent levels of transcripts are observed under PBS and naïve conditions, in contrast to what is observed upon IL1B treatment. O4+ cells from naïve mice: *n* = 6 at P3, *n* = 6 at P5, *n* = 6 at P9; O4+ cells from PBS-injected mice: *n* = 2 at P5; O4+ cells from IL1B-injected mice: *n* = 2 at P5. *Mbp* serves as a positive control of the myelination process that is expected to occur in P9 in control conditions.

The profiles of transcriptomic disturbances induced by neuroinflammation displayed 4 main clusters of genes sharing the same profile dynamics within one given cluster (C1–C4; Fig. 2A, B). First, the maximal contrast of intensity in disturbances in gene expression was observed between profiles at P5 after IL1B treatment and those at P10 after PBS injections, indicating that the expression levels of these genes were going in the opposite direction in response to neuroinflammation, compared to what happened along the normal developmental course (at least for clusters 1 to 3; Fig. 2A, B). Second, exposure to neuroinflammation from P1 to P5 resulted in expression levels at P10 (P10-IL1B) that were intermediate between those in P5-PBS and P10-PBS samples, reflecting that the transcriptomic changes that normally take place during the maturation process of OPCs were incomplete (at least for cluster 1–3; Fig. 2A, B), which was indicative of maturation blockade, as expected in our model (see also the more detailed analysis of cluster 2 and 3, below).

Gene cluster C1 corresponded to genes that were mostly downregulated along the physiological (unstressed) OPC maturation process between P5 and P10 (compare the PBS columns at P5 and P10 in Fig. 2A). In response to neuroinflammation, the vast majority of cluster C1 genes represented those which were the most strongly upregulated at P5 (220 on 262 genes; Fig. 2A). These genes were still active, but in a milder manner at P10 (Fig. 2A). Strikingly, our GO analysis of the C1 genes pinpointed out the immune system and inflammatory response in the top 5 most statistically significant pathways (DAVID 6.8; Fig. 2B; Table S2). We then analyzed: (1) the physiological expression of genes belonging to C1 in control P5 O4+ cells; (2) their upregulation in response to neuroinflammation.

### OPCs intrinsically and developmentally produce cytokines and chemokines in normal conditions

Because, it was unexpected to find genes of the immune/inflammatory pathways intrinsically expressed under uninflamed control conditions, we monitored the dynamics of their expression in MACS-isolated O4+ cells from pups at P3, P5, and P10, by RT-qPCR, to find out whether these genes presented dynamic expression profiles along the OPC maturation trajectory. Indeed, we detected cytokine and chemokine mRNA in O4+ cells in control conditions (Fig. 2C, black curves). We found that cytokine and chemokine mRNA levels were highest at P3 and, then, significantly decreased, in a stage-dependent manner along the maturation process, between P3 and P10 (Fig. 2C). These results were in line with what we observed in our microarray analyses (compare P5-PBS to P10-PBS for C1, Fig. 2A). To exclude that our control conditions (PBS i.p.) could, per se, constitute a stress, which would be responsible for induced expression of cytokine and chemokine genes, we compared MACS-isolated O4+ cells from naïve (non-injected) and PBS-injected pups at P5, in RT-qPCR experiments. We observed similar levels of cytokine and chemokine mRNAs in naïve pups, compared to PBS-treated ones (Fig. 2D). This shows that PBS intraperitoneal injections are not responsible per se for the constitutive cytokine and chemokine mRNA levels physiologically observed at P5.

**Fig. 3:**
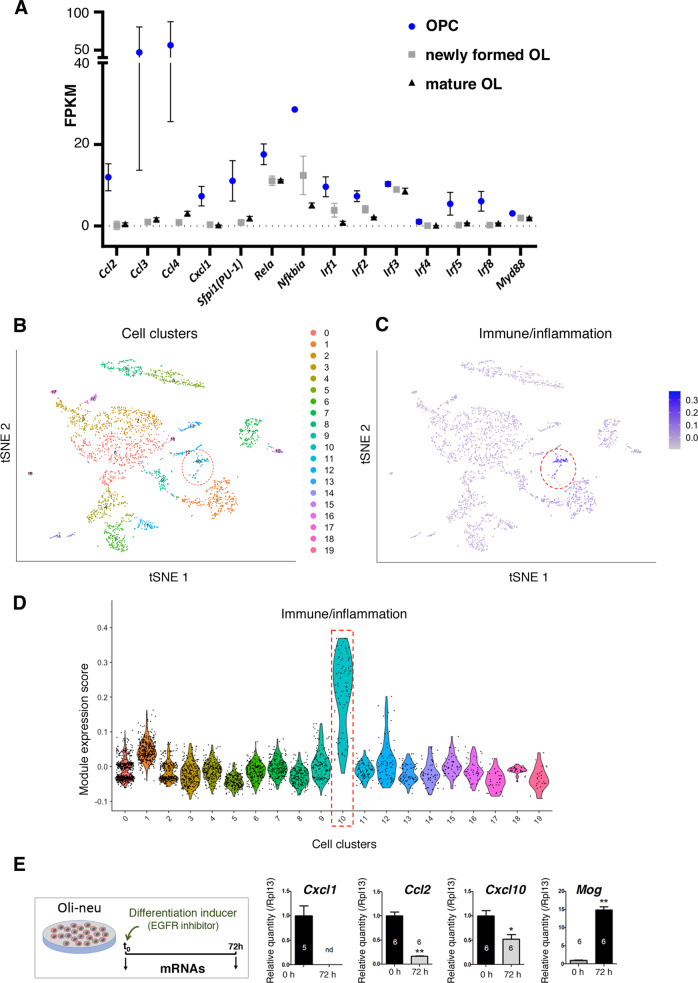
Data mining analyses of the expression of genes of the immune/inflammatory pathways by RNA-Seq in isolated oligodendroglia populations and single-cell RNA-Seq from whole mouse cortices and cytokine and chemokine gene expression in the oligodendroglial cell line Oli-neu (See Fig. S1). **A** Expression of genes of the immune/inflammatory pathways, in OPCs, newly formed oligodendrocytes (OLs) and mature OLs. (RNA-Seq dataset from [13]). These cell populations were purified using transgenic mice carrying immunofluorescent reporter of and depletion of other cells types. Their transcriptome was analyzed by RNA-Seq at P17. Here, the genes investigated belong to gene cluster 1 identified in O4+ cells (Fig. 2A) and include cytokines and chemokines and transcription factors of the immune/inflammatory pathways. FPKM: Fragments Per Kilobase Million. **B** t-distributed stochastic neighbor embedding (t-SNE) plot of twenty major cell populations identified in the control sample (P7 cortical tissues) single-cell RNA-Seq dataset by Jin et al. [14]. Each dot represents a single cell. Clustering was done using Seurat4. Cluster 10 corresponds to Pdgfra+*/*Olig2+ cells, and therefore to OPCs, and is indicated by the red dotted circle. **C** Genes, which belong to the TOP5 GO-terms of the gene cluster C1, show the highest expression in the *Pdgfra*+***/****Olig2*+ cells that correspond to cluster 10. Feature plot showing the distribution of module scores as calculated by Seurat’s AddModuleScore function. Names of the corresponding genes (see also Table S3): *Hif1a, Pdgfra, Cxcl5, Hmgb1, Il17d, Il7, Tnfaip6, Traf6, Acp6, Adam9, Agtr1a, Ampd3, Ankrd17, Arhgap35, Atm, Bves, C2cd4a, Calcrl, Cd1d2, Cd3d, Chd2, Chd7, Chrna4, Cited1, Clock, Cnot4, Cr2, Cyld, Dennd1b, Dock10, Duoxa2, Eif2ak1, Ercc1, Ercc2, Etv4, Fbxo7, Flt3l, Gdnf, Gpr171, Itgav, Klre1, Ldlr, Lrp1, Lrtm2, Masp2, Med23, Ncor1, Nhej1, Nkx2-1, Oas3, Orai1, Pde4b, Pou1f1, Pou4f1, Prkcq, Prrc2c, Ptpre, Ptprz1, Raf1, Rgcc, Rrs1, Rtkn2, Ryk, Sema3d, Sox6, Src, Srp54b, Susd4, Tet2, Tmem176b, Trim11, Trim62, Trpv4, Tspan6, Ufl1, Unc5c, Vav3, Zcchc3, Itgb8, Ddx17, Ep300, Ccdc134, Ifitm7, Masp1, Il1rap, Sema5b, Dscam, Dll1, H2-T3, Tnfsf9, Gpr17, Dcc, Socs6, Rbm14, Ppp1r14b, Zfp950*. Cluster 10 is indicated by the red dotted circle. **D** Violin plot showing the distribution of the module expression score for the same gene set as (**C**), in each cell cluster shown in (**B**). Cluster 10 corresponds to *Pdgfra+/Olig2+* cells, and therefore to OPCs exhibits, the highest gene expression levels. **E** The oligodendroglial cell line Oli-neu recapitulates the constitutive expression of cytokine and chemokine genes at the immature state as well as its downregulation upon differentiation (see also Fig. S1E). (Left panel) Differentiation protocol of the Oli-neu cell line triggered by PD174265, a potent, cell-permeable inhibitor of the tyrosine kinase activity of the epidermal growth factor receptor (EGFR; [56]; see “Materials and methods”). (Right panel) RT-qPCR analyses in the Oli-neu cell line before or after 72 h of differentiation. This in vitro paradigm displays the same pattern as O4+ cells, in terms of constitutive expression of genes encoding inflammatory mediators at the immature (undifferentiated) state and its decrease when maturating to myelin-producing oligodendrocytes. Numbers of independent experiments are indicated on the graph bars. *p < 0.05; **p < 0.01. nd not detected.

To further address the possibility that OPCs were intrinsically able to synthesize cytokine and chemokine mRNAs, we performed two data mining studies. First, we examined RNA-Seq data in glial and neuronal populations from mouse cortices [13], which included maturing OPCs/OLs at P17. These populations were independently purified using two different protocols to ensure that they did not affect gene expression differently. We found that OPCs, newly formed OLs, and mature OLs constitutively expressed genes of the immune/inflammatory pathways (Fig. 3A) that belonged to gene cluster C1 as described in Fig. 2A. Moreover, we found that the levels of these transcripts were higher in OPCs than in newly formed OLs or matured OL (Fig. 3A), which corroborated our findings that genes of the immune/inflammatory pathways are downregulated along the OPC maturation process (Fig. 2C, D). Second, we analyzed published single-cell transcriptomic data from whole neocortices of control mouse pups at P7 [14]. Among the 19 clusters of cell populations identified (Fig. 3B and Fig. S1A), we identified cell cluster 10 as containing OPCs/immOLs, on the basis of *Olig2* and *Pdgfra* expression (Fig. 3B and Table S3). We found that genes belonging to the top5 GO-terms immune-inflammatory pathways of gene cluster C1 (Fig. 2B), were expressed at high levels in this cluster 10 corresponding to OPCs/ImmOLs (Fig. 3C, D). This was striking because we expected that many genes of the immune/inflammatory pathways would be downregulated at P7 (compare PBS samples at P5 and P10 in the C1 gene group in Fig. 2A, C), and, actually, we found that the expression of some of these cytokines and chemokines was very low at P7 in this OPC/ImmOL-like cell population (Fig. S1B). Nevertheless, our analyses demonstrate that the *Pdgfra+/Olig2+* cell cluster 10 can intrinsically express other genes of the innate immune/inflammatory pathways, indicating that this pathway is active in OPCs/immOLs (Fig. 3C, D, Fig. S1C, D, and Table S3). Of note, in the study by Jin et al., cell populations were neither purified nor enriched, but analyzed from whole cortices ([14] see “Discussion”).

Importantly, we could recapitulate the constitutive expression of cytokine and chemokine genes observed in P5 O4+ cells in a murine oligodendroglial cell line, Oli-neu, under normal conditions (Fig. 3E). Moreover, using two distinct protocols of differentiation, we showed that this constitutive expression was decreased upon Oli-neu differentiation, in a manner similar to what was observed during the maturation trajectory of O4+ cells (Fig. 3E and Fig. S1E).

Altogether, our results reinforce the fact that the synthesis of molecules of the immune/inflammatory pathways in O4+ cells is neither due to contamination by other cell types during their purification, nor to the stress potentially inflicted by the cell purification protocol or by i.p.injection, but represents an intrinsic property of OPC/immOLs. Altogether, these data demonstrate that uninflamed P5 O4+ cells intrinsically transcribe cytokine and chemokine genes at an early OPC stage (P3), and that the expression of these genes is gradually downregulated during their maturation process between P3 and P10, in a physiological and developmental manner.

### OPCs intrinsically induce cytokines and chemokines in response to neuroinflammation

**Fig. 4:**
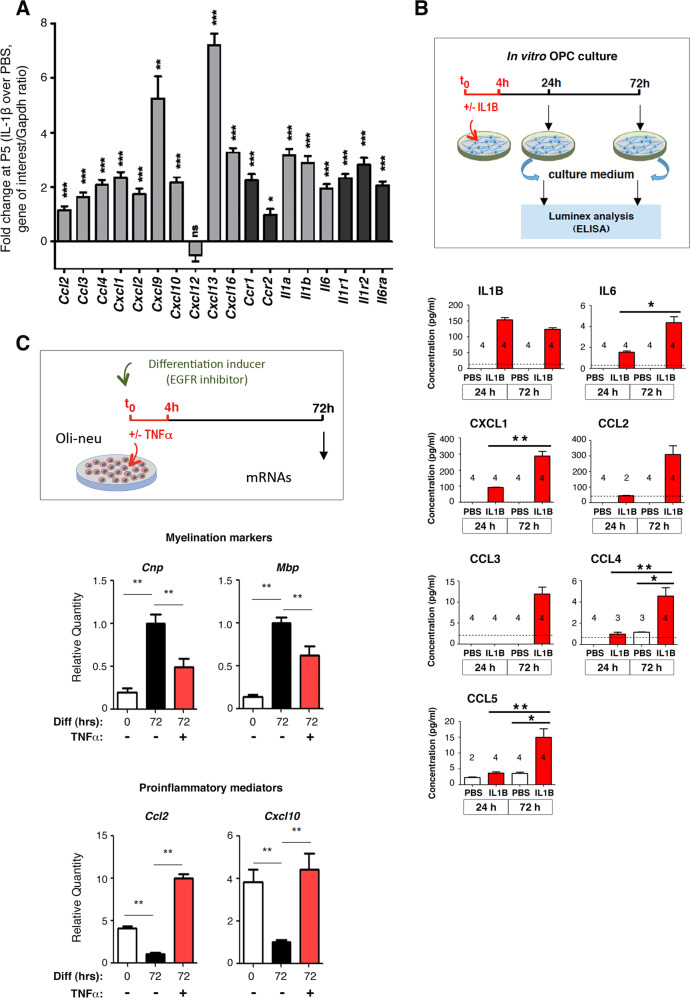
O4+ cells intrinsically synthesize molecules of the Immune/inflammatory pathways in response to neuroinflammation (See Fig. S2). **A** Validation of the induction of gene expression for members of the GO-term “immune system and inflammatory response pathways” by neuroinflammation, in O4+ cells at P5, using RT-qPCR. Number of independent experiments: n = 8 for all genes, except for *Cxcl9*, *Cxcl10*, and *Il1r1* (*n* = 7). *p < 0.05; **p < 0.01; ***p < 0.001. Gray bars: ligands; black bars: receptors. **B** In vitro cultured OPCs intrinsically secrete mediators of the immune and inflammatory pathways in response to inflammatory stimulus. (Upper panel) Experimental design for ex vivo OPC culture, inflammatory exposure (IL1B), and differentiation (see Material and Methods). (Lower panels) Protein detection and quantification (pG/mL) of the expression of interleukins (IL1B, IL6), cytokine C-C Motif Chemokine Ligand 2, 4, and 5 (CCL2, CCL3, CCL4, and CCL5) and chemokine C-X-C Motif Chemokine Ligand 2 (CXCL2) by Luminex. CTR, PBS exposure; IL1B, IL1B exposure. The dotted line represents the limit of detection for individual proteins in the assay. The numbers of independent experiments performed for each plot are indicated on each plot. *p < 0.05; **p < 0.01; ***p < 0.001. **C** Upregulation of genes of the immune/inflammatory pathways, in the oligodendroglial cell line, Oli-neu, and downregulation of the myelination program, upon inflammatory stimulus. Treatment of the Oli-neu cell line by TFN-alpha reproduces the disturbances in gene expression that we observed in isolated O4+ cells upon in vivo neuroinflammation. (Upper panel) Experimental design. (Lower panels) RT-qPCR analyses for myelinations markers and proinflammatory mediator genes *Cnp* and *Mbp*, and *Ccl2 and Cxcl10*, respectively. *n* = 6 independent experiments. Nonparametric *t*-tests; **p < 0.01. Time 0: undifferentiated cells.

We then focused on the induction of genes belonging to the C1 gene cluster by neuroinflammation (Fig. 2A). Using RT-qPCR, we confirmed the induction of genes encoding known mediators of the immune/inflammatory pathways by neuroinflammation in O4+ OPCs at P5 (in independent samples, with regard to those analyzed using microarrays): cytokines, chemokines, interleukins, and their receptors (Fig. 4A). Using Luminex protein detection, we also detected increase in corresponding proteins in the medium of in vitro (not MACSed) cultivated OPCs submitted to IL1B exposure (see Materials and Methods; Fig. 4B), in line with our previously observations [12]. We also demonstrated by RT-qPCR experiments that neuroinflammation, by markedly inducing the upregulation of genes of the innate immune/inflammatory pathways at P3 and P5, counteracted the physiological downregulation of these genes that should physiologically take place at P5 (Fig. 2C). Importantly, we recapitulated these findings in the oligodendroglial precursor cell line, Oli-neu, by demonstrating that the Oli-neu cell line had the intrinsic ability to upregulate genes of the immune/inflammatory pathways in response to a proinflammatory stimulus, TNF alpha (Fig. 4C).

The fact that the oligodendroglial cell line Oli-neu recapitulated the neuroinflammation-induced expression of immune/inflammatory pathways of the C1 cluster was strongly indicative that what we observed in inflamed O4+ cells at P5 was not due to contamination by other cell types. Notably, this hypothesis was also ruled out by a panel of diverse approaches, by which we evaluated the purity of the O4+ cell populations. Globally, we showed that specific markers of microglia and astrocytes are unexpressed in the isolated O4+ cell population. Using either transcriptomic microarray data from CD11B+ microglial cells, obtained from the same animals as the O4+ cell populations of this study, or RT-qPCR, we demonstrated that the upregulation of cytokine and chemokine transcripts of the C1 gene cluster was greater in P5 O4+ cells than in microglia or astrocytes (Fig. 4A, Fig. S2; for detailed description of our data, please see Supplemental information related to Fig. S2).

Together with the recapitulation of our data in the oligodendroglial Oli-neu cell line, which cannot be contaminated by other glial or microglial cell types, our results strongly support the notion that the neuroinflammation-induced upregulation of genes of the immune/inflammatory pathways is an intrinsic property of O4+ cells in the developing cortex at P5.

### Primed chromatin landscape in the C1 genes of the immune/inflammatory pathways shapes the neuroinflammatory response in O4+ OPCs

The fact that the major transcriptomic perturbation triggered by neuroinflammation impacts the immune/inflammatory pathways in O4+ cells at P5 is striking. So is the tight control of the developmental regulation of these genes as an intrinsic component of oligodendrocyte maturation between the late OPCs and immOLs. Our results suggest that the constitutive property of O4+ OPCs to synthesize cytokines and chemokines in a stage-specific manner during their maturation process might constitute an entry point for the impact of neuroinflammation in maturating O4+ cells.

This prompted us to investigate the chromatin landscape of the 1,250 upregulated genes (called “UP”; identified by using NCBI gene ID) and of the 451 downregulated genes (“DOWN”) upon neuroinflammation, respectively. For this, we investigated the impact of neuroinflammation on the integrity of the chromatin landscape in O4+ cells using ATAC-Seq at P5 ([15]; *Assay for Transposase-Accessible Chromatin with high-throughput sequencing*). Using the bioinformatics workflow described in Fig. S3A, including the MACS2 and EdgeR software tools ([16, 17] see “Material and methods”), we obtained an average of 72 million Tn5 transposase-integrated mapped reads per sample, representing a total of 213,246 statistically significant peaks (MACS2; FDR < 0.05; Tables S4, S5; Dataset SRA PRJNA540409). The number of reads, which reflects chromatin accessibility, was determined for each sample in the 213,246 peaks. Analysis of the insert size distributions showed the expected nucleosome-induced pattern and 10.4 bp periodicity with good consistency across samples, an indication of high data quality (Fig. S3B; [18]).

**Fig. 5:**
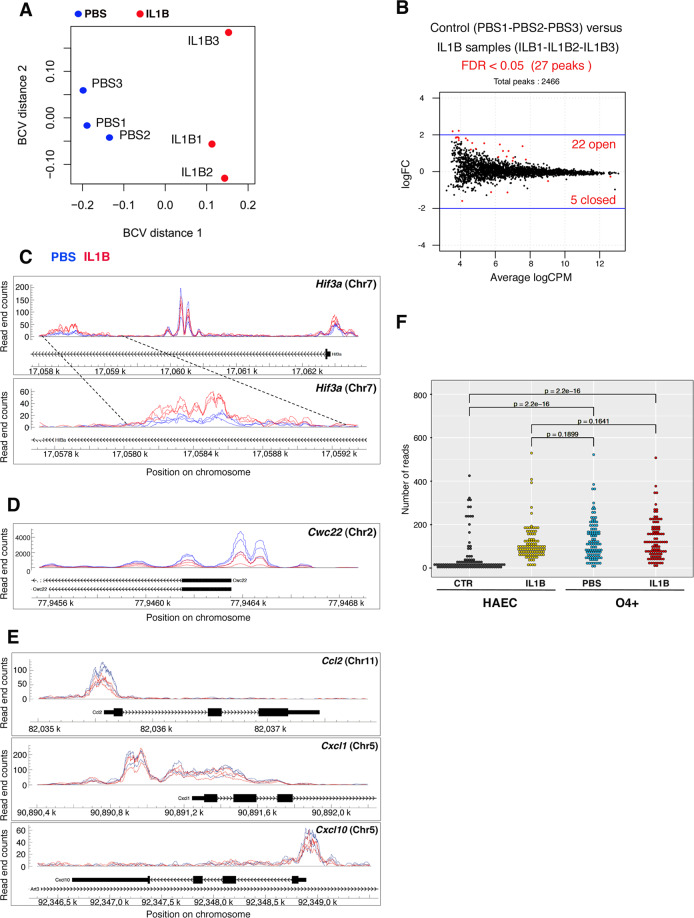
Pre-existing, primed, open chromatin landscape in P5 O4+ cells, in physiological conditions (see Figs. S3, S4; see Table S5). Analysis of regions lying within ±8 kb, around the TSS of genes of the immune/inflammatory pathways (gene cluster C1) that are upregulated in O4+ cells at P5, in response to neuroinflammation. **A** Multidimensional Scaling plot of distances between the 3 PBS samples and the 3 IL1B samples, of the ATAC-seq peaks associated to UP genes, using EdgeR. Distances between samples correspond to biological coefficient of variation (BCV), that represents the biological variation between neuroinflammation-exposed brains and control brains. IL1B samples are only poorly separated from PBS samples, indicating that the chromatin accessibility is mainly unchanged by neuroinflammation in the genes of the immune/inflammatory pathways in O4+ cells. **B** Scatter plot representing the dispersion (fold change) of peaks in relation to the number of tn5 cut*s* per million (logCPM), for each individual analyzed peak located ±8 kb around the TSS of UP genes after neuroinflammation, across the 3 PBS samples and 3 IL1B samples. The 27 peaks showing significant differences between IL1B and PBS conditions are indicated in red, with 22 peaks corresponding to increased (“open”) and 5 peaks to decreased (“closed”) chromatin accessibility, with FDR < 0.05, respectively. **C** Examples of peaks showing increased or unmodified chromatin accessibility: (Upper panel) the peaks located at the most downstream position in the *Hif3a gene* (*Hypoxia-inducible factor 3 alpha subunit*) show increased chromatin accessibility in neuroinflammation context. (Lower panel) Magnification of this region. In contrast, peaks located in the middle of the *Hif3a* gene show no significant changes in chromatin accessibility (see the upper panel). **D** Example of peak showing reduced chromatin accessibility: peaks within the *Cwc22* gene (encoding the spliceosome-associated protein 22). **E** Examples of peaks in genes encoding cytokines and chemokines, showing no statistically relevant modification of chromatin accessibility. **F** Cross-species comparison confirms global similarities in the chromatin landscapes of HAEC and unstressed O4+ cell datasets. (See Table S8) Comparison of 100 peaks which were found to be differential in the HAEC (public) dataset upon IL1B treatment, among all (7739) matched peaks between human (HAEC) and mouse (O4+ cell) datasets. Reads were normalized for each set of peaks against the total number of reads present in all matched peaks and converted into reads per million. Distributions were compared using a one-sample Wilcoxon rank test.

We then explored the ATAC-Seq peaks that were associated to the UP and DOWN genes, based on proximity to their transcription start site (TSS), to maximize the chance of attributing peaks to relevant genes: we reasoned that, peaks corresponding to opening or closing of the chromatin and lying within ±8 kb around a TSS (corresponding to 43,209 ATAC-seq peaks) were most likely to contribute to the regulation of the expression of the corresponding neighboring gene. Using the EdgeR MDS function, we performed multidimensional scaling plot of distances between the 3 PBS samples and the 3 IL1B samples and observed that principal BCV (Biological Coefficient of Variation) distance 1 separated samples from control and neuroinflammation-exposed O4+ cells (Fig. 5A). We found that the 1250 UP genes (Fig. 1C) were associated with 2466 peaks located within ±8 kb around a TSS—peaks, which corresponded to 950 different unique gene names (annotation tool *NCBI gene ID*; Table S6). Among the peaks present in ±8 kb around the TSS, we identified 27 regions that were either open or closed at P5, in a statistically relevant manner in response to neuroinflammation (22 and 5 peaks, respectively; FDR < 0.05; Fig. 5B; Fig. S3C; Table S7). In contrast, the 451 DOWN genes that were with associated with 1040 peaks corresponding to 377 different genes names, did not display differential chromatin accessibility in response to neuroinflammation.

Among the 27 regions associated with differential opening and closing of chromatin conformation, 19 were involved in the immune system and inflammatory response pathways: *Cd14, Chst2, Cwc22* (illustrated in Fig. 5D), *Hmha1, Ifit3, March1, Mcoln2, Mpeg1, Nckap1l, Phyhd1, Pu.1-Sp1, Rrbp1, Slamf1, Slfn2, Slc15a3, Tlr1, Tnfsf14, Tnfrsf12a, Trim30d* (in red in Table S7). Interestingly, *Hif3a*, a gene identified in models of inflammation (see discussion), was also present in the list (illustrated in Fig. 5C, D) for illustration of peaks showing differentially open and closed peaks for *Hif3a* and *Cwc22*, respectively. This means that the most prominent pathways concerned by changes in chromatin accessibility is represented by the immune system/inflammatory response pathways. Nevertheless, among the 262 genes of this C1 gene cluster, the vast majority of them exhibit no changes in chromatin accessibility (almost 90%).

We verified that these results were not due to contamination with microglia. We first investigated the chromatin status of cytokine and chemokine genes in our MACS O4+ OPC population, compared to microglia. The corresponding microglia-specific genes displayed inaccessible chromatin conformation in O4+ cells, in contrast to that of chromatin regions of OPC-specific genes (please see Fig. S4A, B, as well as detailed description of the analyses in Supplementary information related to Fig. S4A, B). We thus concluded that contamination of MACS-isolated O4+ cells by microglia, if any, is only very minor in this study, as it is undetectable in our ATAC-Seq experiments. Therefore, such contamination cannot account for the open chromatin status detected in cytokine or chemokine genes in the MACS-isolated O4+ cell populations. This is also coherent with the differential behaviors in gene expression of the genes of immune/inflammatory pathways belonging to gene cluster C1 that we found between microglia and O4+ cells, in our transcriptomic analyses (Fig. S2B, C).

To further challenge our assumption that, in O4+ cells at P5, the chromatin of neuroinflammation-induced C1 genes constitutively exhibited open conformation prior to IL1B treatment, we formulated and performed an additional analysis, taking advantage of an existing public ATAC-seq dataset from a similar control versus treatment study. This study used IL1B stimulus on human adult aortic endothelial cells (HAECs) isolated from aortic trimmings of donor hearts ([19]; “HAEC dataset”; NCBI Gene Expression Omnibus; accession no: GSE89970). Through a cross-species comparison, our aim was to explore global chromatin landscape similarities (or lack thereof) between both control and IL1B-treated adult HAECs and neonate O4+ cells. Both datasets were limited to chromatin regions annotated with matching 1-to-1 gene orthologs and located within ±2 kb of a TSS, as described in Material and Methods. In total, we were able to match 7739 peaks between the two datasets, including 100 regions that were found to be different between the control and IL1B conditions in the HAEC dataset. Subsequent cross-comparison of these regions reveal no significant difference in chromatin accessibility between either uninflamed or inflamed O4+ cells and IL1B-treated HAECs. In contrast, both uninflamed and inflamed O4+ cell samples showed significant differences (*p* value < 10^−15^) to the HAEC control sample (Fig. 5F and Fig. S4C; Table S8). These results confirm that uninflamed O4+ cells exhibit a profile of chromatin accessibility resembling that acquired by HAECs upon IL1B treatment and similar to that of inflamed O4+ cells. This analysis thereby reinforces our findings that genes of the immune/inflammatory pathways belonging to gene cluster C1 are in an open chromatin conformation and active both in uninflamed and inflamed O4+ cell populations.

In summary, the genes that represent the most prominently dysregulated cluster of genes upon exposure to neuroinflammation (C1 cluster), belong to innate immune system/inflammatory response pathways and are associated to two different chromatin behaviors: (1) only a limited number of the top 5 genes (19 genes out of 260) showed differential chromatin accessibility upon IL1B exposure; (2) the majority of these genes undergo no major changes in chromatin accessibility, as they are already in an open conformation and already transcribed under control conditions. This indicates that the most prominent transcriptomic changes triggered by neuroinflammation in O4+ cells at P5, occur in a primed epigenomic landscape setting the stage for these major perturbations.

### Identification of combinatorial transcription factor-binding belonging to the immune system and inflammatory pathways

**Fig. 6:**
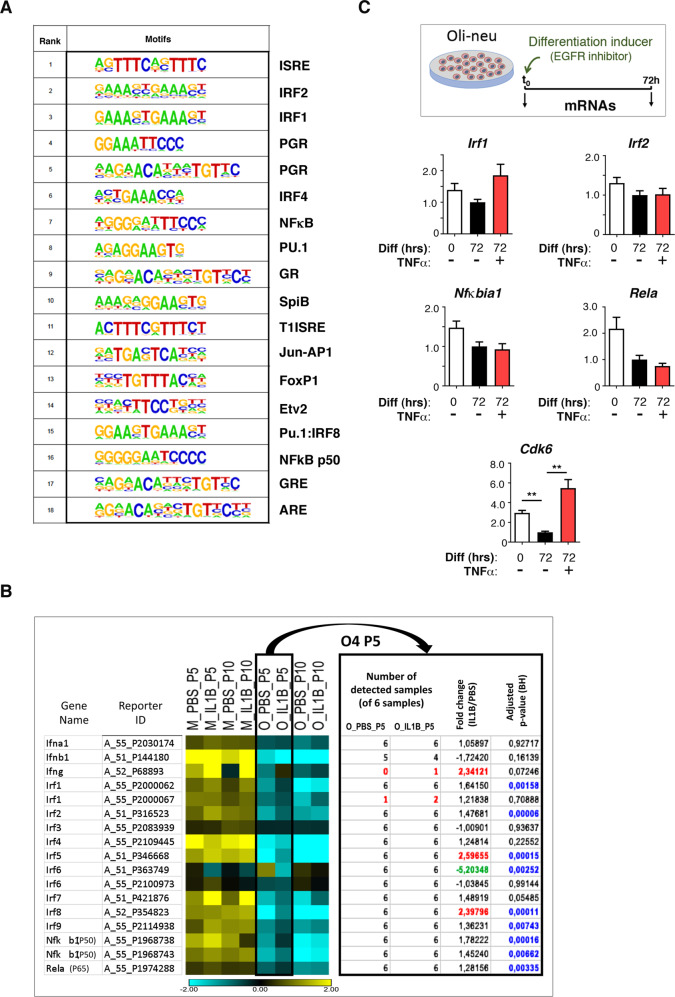
TFBS motifs identified in significant ATAC-seq peaks associated with cluster C1 genes and expression of the corresponding TF genes in O4+ cell populations and the Oli-neu cell line. **A** TFBS motif analysis reveals the presence of TF binding motifs of the immune/inflammatory pathways in significant ATAC-seq peaks that are adjacent to the upregulated genes (UP) correspond to TF of gene cluster C1. TFBS were identified using HOMER known motifs. (See also Fig. S5). **B** Heat map illustrating the expression of TFs of the IRF and NFκB families in O4+ cell populations. Microarray data from microglia [9]; *n* = 3 PBS samples and *n* = 3 IL1B samples isolated from the same cortices and at the same stage is given for comparison. M: Microglia. O_P5: O4+ cells at P5. O_P10: O4+ cells at P10. **C** The oligodendroglial Oli-neu cell line recapitulates the expression of TF genes of the immune/inflammatory pathways in physiological and inflammatory conditions. As in Fig. 4C. *Cyclin-dependent kinase 6* (*Cdk6*) mRNA levels were analyzed in parallel.

We thus investigated the putative involvement of transcriptional regulators as primary mediators of alteration in gene expression in this open chromatin landscape in O4+ cells at P5. We searched for enrichment in transcription factor-binding sites (TFBS) using HOMER and known motifs, in the ATAC-Seq peaks adjacent to differentially “UP” genes belonging to cluster C1. For this, we focused on ATAC-Seq peaks located within a distance of ±8 kb relative to the TSS of these genes. We found that motifs for members of the IRF (interferon-regulatory factor) family appeared at the top of the list, with the strongest scoring results, as well as motifs for the NFκB family members and the composite site PU.1-IRF8 (Fig. S5A and Fig. 6A). A list of similar motifs was found with comparable scoring results and *p* values, in the peaks adjacent to all dysregulated genes (up- and downregulated genes (termed “ALL”); Fig. S5B), which suggests that the most prominent perturbations of the O4 cell transcriptome are driven by TFs involved in the immune/inflammatory pathways. No motif enrichment was found in DOWN genes.

Interestingly, by exploring the transcript dynamics of these TFs, we found that the genes encoding members of the IRF and NFκB families corresponding to these motifs were all expressed in uninflamed OPCs (Fig. 6B). Of note, their transcripts were present at lower levels in OPCs than in MG, except for *Irf3*, *Irf6* and *Rela*, which displayed comparable levels in these two cell populations. Among these factors, *Irf5* and *Irf8* were significantly upregulated by neuroinflammation in OPCs, whereas *Irf6* was downregulated (Fig. 6B). We also demonstrated that Oli-neu constitutively expressed members of the IRF and NFκB families, which reinforced our findings in O4+ cells (Fig. 6C). Moreover, we found that these TF mRNA levels were decreased both in P10 O4+ cells and upon Oli-neu differentiation, which is also in line with the stage-dependent downregulation of cytokines and chemokine genes during OPC maturation between P5 and P10 and along Oli-neu differentiation (Fig. 6B, C).

**Fig. 7:**
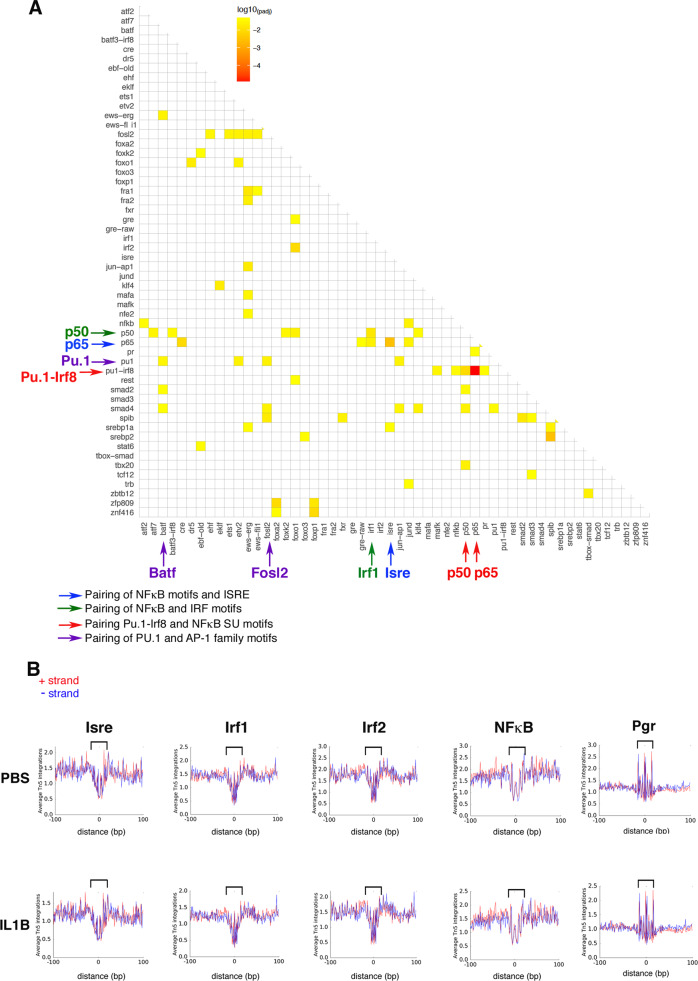
Chromatin accessibility footprints at TF motifs reveals the major involvement of TFs of the immune and inflammatory pathways in O4+ cells. **A** Co-localization of pairs of TF binding motifs in ATAC-Seq peaks of upregulated genes. Heat map of the paired motifs located in the 2319 ATAC-Seq peaks located within ±8 kb from the TSS of 1266 upregulated genes (corresponding to 886 different gene names), identified using paired-m 0; **A** Heat map of the paired motifs located in the 2319 ATAC-Seq peaks located within +/- 8 kb from the TSS of 1266 upregulated genes (corresponding to 886 different gene names), identified using paired motif analysis. TF names from MEME nomenclature on both axes. Statistically relevant co-occurrence of two different TFs is indicated by yellow to orange squares (multiple testing corrected log10 p-value). Examples of TF pairs are indicated IRF1-p50; ISRE-p65; PU.1-IRF8-p65; PU.1-iRF8-p50; PU.1-BATF; PU.1-FOSL2; *ISRE*-p65; PU.1-IRF8-p65; PU.1-iRF8-p50; PU.1-BATF; PU.1-FOSL2. Color code (TF names and arrows): red, pairing of PU.1-IRF8 with NFκB subunit binding motifs; purple, pairing of PU.1 with AP-1 family binding motifs; blue, pairing of NFκB binding motifs with *Isre* (IFN-stimulated response element); green, pairing of NFΚB with IRF binding motifs. **B** Average footprint profiles at TF binding motifs plotted at base pair resolution, using the Wellington software [21], located within accessible chromatin *loci* adjacent to any differentially regulated genes (down or upregulated) for the following TF or binding sites: *Isre*, IRF1, IRF2, NFκB in PBS-treated or IL1B-treated samples, indicating that these TFs might already bind DNA. Typical footprint profiles coincide with the center of the canonical motif for a given TF (indicated by brackets). Footprints showing occupancy by IRF1, IRF2, *Isre*, and NFκB of corresponding binding motifs are illustrated. Controls: average footprints showing sharp internal spikes are suggestive of transposase insertion bias at PGR-factor-binding site. Red and blue curves: + and − strand, respectively.

We suspected that some of these TFs might work in partnership with each other [20]. We thus investigated the occurrence of paired motifs in the peaks located in ±8 kb regions around the TSS of differentially regulated genes, using the PMET tool (“Materials and methods”). The analysis of peaks corresponding to DOWN genes did not point any paired-motif enrichment out, compared to random occurrence in all peaks. In contrast, our analysis of ATAC-Seq peaks associated with UP genes revealed the existence of paired TFBS motifs, with marked involvement of TFBS from the IRF family, Pu.1/Spi1, *Isre* (Interferon-Stimulated Response Element), NFκB, and AP-1 family (Fig. 7A; Table S9). We then investigated whether the occurrence of these motifs was accompanied by the binding of TFs to DNA in O4+ cells. For this, we used the Wellington algorithm, which is highly accurate in inferring protein TF-DNA interactions [21], through the detection of footprints corresponding to occupied TFBS and of their motif content. Figure 7B illustrates the average footprint profiles for IRF1, IRF2, IRSE and NFκB that are located within significant ATAC-Seq peaks adjacent to differentially regulated genes and produced for the top 6, high-ranked, HOMER motifs described in Fig. 6A and Fig. S5. The dip in the number of reads at the center of the sharp average profile (indicated by brackets) was indicative of effective TF binding (Fig. 7B). In contrast, PGR (Progesterone Receptor) average footprints exhibited sharp internal spikes, which was suggestive of transposase insertion bias and not of DNA occupancy ([22]; Fig. 7B). There was no evidence for footprints in peaks adjacent to downregulated genes (data not shown). Notably, the search for de *novo* motifs in ATAC-Seq peaks near differentially expressed genes did not reveal statistically relevant motif associated with bona fide average footprints (data not shown). Interestingly, for all these TFs showing footprints and thus clear DNA occupancy, we observed little difference in the average footprint profiles comparing PBS and IL1B samples, indicating that these TFs (or TFs with binding similar motifs) not only bind DNA at the corresponding motifs upon neuroinflammation, but also under uninflamed, physiological conditions (Fig. 7B), in line with the constitutive expression of genes of the Immune/inflammatory pathways in uninflamed O4+ cells, under normal developmental conditions.

### Concomitant dysregulation of genes of the immune/inflammatory, cell cycle and myelination programs by neuroinflammation signs a global cell fate problem in O4+ OPCS

We showed that the upregulation of genes of the immune/inflammatory pathways by neuroinflammation counteracted the physiological stage-dependent decrease of their expression that is observed between P3 and P5 in control animals (Fig. 2C) and upon Oli-neu differentiation (Figs. 3E and S1E and Fig. 4C). In addition, we showed by RT-qPCR experiments that the upregulation of genes of the immune/inflammatory pathways in response to neuroinflammation or in vitro IL1B exposure was concomitant with impaired expression of genes of the myelin program in both systems (Figs. 1B, 2C, and 4C). Importantly, we demonstrated that these opposite and concomitant profiles were also observed at a global level in our microarray analysis (compare cluster C1 to C3; Fig. 2A).

**Fig. 8:**
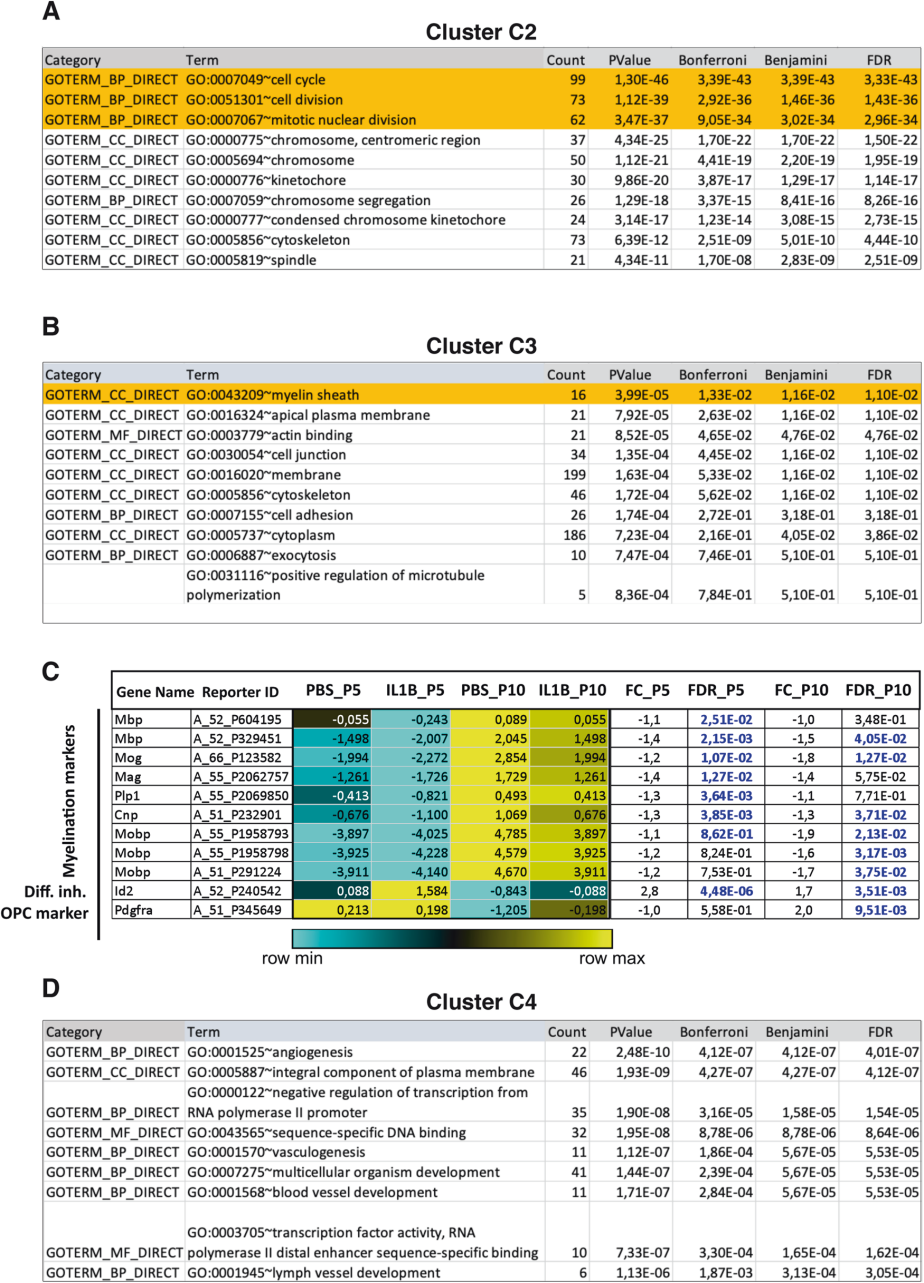
GO analysis corresponding to the TOP-10 genes belonging to clusters C2, C3, and C4, using DAVID 6.8. (related to Fig. 2A). **A** Cluster C2 correspond to genes involved in the regulation of the cell cycle. As expected, in unstressed, normal conditions, these genes are expressed at P5 and downregulated at P10, as OPC maturation progresses and proliferation is reduced (Fig. 2A) This physiological downregulation observed at P10 is markedly counteracted by neuroinflammation, which causes their upregulation at P10. As a result, the expression of these genes is intermediate between their expression levels at P5 and P10 in controls, which might reflect differentiation blockade (this is true for clusters 1, 2, 3). **B** Cluster C3 correspond to genes involved in myelin sheath formation, cell adhesion and cytoskeleton. All the underlying processes are important for the differentiation of OPCs and completion of the myelination program. As expected in normal O4+ cells from unstressed pups, these genes are developmentally upregulated at P10 (Fig. 2A, compare P5-PBS to P10-PBS), and their developmental dynamics of expression is impaired by IL1B injections, as expected in this model of perinatal neuroinflammation, which induces OPC maturation blockade. **C** Expression of genes of the myelination program belonging to cluster 3. All the microarray probes for every gene of the myelination program analyzed here show statistically significative alteration in gene expression. *Id2* (transcriptional inhibitor of oligodendrocyte differentiation) and *Pdgfra*, an OPC marker. Log2 intensities, median-centered. Relative color scheme. **D** Cluster C4 correspond to genes involved in diverse functions, including angiogenesis, vascularization, and integral component of the plasma membrane*.* These functions are important for the myelination process which is very demanding in energy (blood supply) and for the correlative increase in surface of the plasma membrane. Half of these genes is downregulated at P5, and none of these C4 genes are altered in its expression at P10.

By comparing uninflamed to inflamed O4+ cells at P10, it appeared that global gene expression levels in P10-IL1B samples were intermediate between those observed at P5-PBS and those at P10-PBS (compare lane P10-IL1B to P5-PBS and P10-PBS in Fig. 2A). For cluster C1, it signed the upregulation of genes of the immune/inflammatory pathways by neuroinflammation; which counteracted the stage-dependent decrease in the physiological of these genes that is observed between P3 and P5 (Fig. 2C). At P10, the appearance of such intermediate, global gene expression profiles was also observed for C2 and C3 clusters, and corresponded to the deregulation of genes regulating the cell cycle and genes involved in the myelination process, respectively (Figs. 2A, 8A, B; Table S10; Table S11). Whereas the expression of cell cycle genes belonging to cluster C2 should decrease between P5 and P10 (compare P5-PBS and P10-PBS; Fig. 2A), neuroinflammation provokes their upregulation at P10 (compare P10 PBS and P10-IL1B; Fig. 2A; abnormal upregulation of Cdk6 mRNAs was similarly observed in differentiated Oli-neu cells upon TNFα-treatment; in Fig. 6C). Similarly, whereas myelination gene expression should increase between P5 and P10 (compare P5-PBS and IL1B PBS), our examination of the expression profiles of the genes of the cluster C3 revealed that neuroinflammation impairs the myelination program in a global manner by affecting, not only genes encoding components of the myelin sheath itself, but also genes important for oligodendrocyte myelination functions, including cytoskeletal remodeling and cell-cell interactions (Fig. 2A; Fig. 8C; [23, 24]).

Finally, cluster C4 was characterized by global and severe downregulation of genes involved in late OPCs either in angiogenesis, vascularization, or as integral components of the plasma membrane (Fig. 2A; Fig. 8D; Table S12). A tight link has been reported between myelination and angiogenesis in the literature: OPCs display huge metabolic needs during their maturation, in order to produce multiple myelin segments, as well as undergo tremendous increase in plasma membrane surface, processes that rely on appropriate access to blood supply [25]. Yuen et al. [26] and Chavali et al. [27] demonstrated that the OPC display unexpected and intrinsic ability to promote angiogenesis *via* paracrine activity (see Discussion). Therefore, compromised expression of pro-angiogenic genes in inflamed O4+ cells might also contribute to the reduction of myelination capacities observed upon neuroinflammation.

These unbiased genome-wide observations therefore not only reinforce and extend our data obtained in RT-qPCR experiments in O4+ cells and the oligodendroglial cell line Oli-neu, but provide a wide picture of the general impact of neuroinflammation and of the nature of the OPC maturation blockade, immediately and at distance for the insult. Indeed, we demonstrate that neuroinflammation undergone between P1 and P5 induces global cell fate issues in O4+ cell populations at P10. This reveals, at the transcriptomic level, an in-between, “frozen” maturation state, which involves 2420 among the 2771 mRNAs showing differential levels and involving the perturbation of key pathways governing the physiological expression of immune/inflammatory molecules, cell cycle, and myelination process (Fig. 2A).

## Discussion

The way neuroinflammation impacts the OPC maturation process, further leading to DWMI, is unclear. Here, through unbiased approaches, we provide global profiling of the impact of neuroinflammation and uncover that neuroinflammation elicits genome-wide signs of global cell fate issues that affect O4+ cell populations. This includes an overall impairment of the myelination program, as expected from previous studies [3, 7, 8] and also the dysregulation of unexpected key pathways: (1) the striking upregulation of genes of the immune/inflammatory pathways in O4+ cells at P5 and (2) the overexpression of genes of the cell cycle pathway at P10, a stage at which cell proliferation has ceased.

First, one of our most important findings is that uninflamed O4+ cells at P5 have the remarkable capacity to intrinsically and physiologically express numerous genes of the innate immune/inflammatory pathways during murine cortical development, at stages equivalent to the third trimester of pregnancy. These genes exhibit constitutive chromatin accessibility prior stress, thereby providing a primed epigenetic landscape that shapes the response to neuroinflammation, which acts by boosting their expression. In that sense, the epigenome priming of O4+ cells thus empowers neuroinflammation to induce an innate immune-like state at P5, by which the expression of immunomodulators, instead of being transient, persists in an abnormal manner. Interestingly, in a disease context of multiple sclerosis and demyelination, in the adult brain, OPCs make a transition to an immune-like state [28].

The developmental expression of cytokine and chemokine genes, which is thus associated with a pre-wired, primed, open chromatin landscape is highjacked by neuroinflammation that provokes abnormal overexpression of these genes at a stage (P5) it is normally downregulated. This thus occurs with limited effects on chromatin accessibility. Such a mechanism is reminiscent of what happens in response to cellular stress, involving the heat shock pathway driven by the Heat Shock transcription Factors, which is “pre-wired” by the pre-existing chromatin architecture (HSFs; [29, 30]). A recent study on multiple sclerosis susceptibility by Meijer et al. [31] also reported epigenomic priming immune genes in both human and murine oligodendroglia at immune genes, in the adult brain. During brain development, the impact of neuroinflammation therefore seems to also rely on epigenomic priming as it is the case in the adult brain and might represent share features with other stress responses.

Our demonstration of the intrinsic property of OPCs to synthesize molecules of the immune/inflammatory pathways is strengthened by the following considerations. One important interest of our study is that we used purified populations of OPC/immOLs, based on the O4 marker to capture the global disturbances induced by neuroinflammation. To do so, we carefully assessed the purity of our isolated populations by different means and at each step of our study, as summarized in Figs. S2, S4 and related Supplementary information. Therefore, the assessment of the purity and functionality of this isolated O4+ cell population is built on a corpus of arguments that altogether lead to the conclusion that O4+ cells at P5 are able to constitutively synthesize molecules belonging to the immune/inflammatory pathways and is totally coherent with recent findings in the literature. This includes studies of patients affected by multiple sclerosis (MS) or in in vivo models of experimental autoimmune encephalomyelitis (EAE; reviewed in [31, 32]. Our results are also in line with previous results showing that OPCs, derived in vitro from neurospheres, can activate cytokine genes in an EAE model [32, 33]. In addition, our findings are in line with single-cell transcriptomic data from whole brain that point out that OPCs express genes of the immune/inflammatory pathways ([14 and our data mining analyses). The possibility that whole-tissue cell dissociation could have triggered stressful conditions is unlikely because the authors validated their findings by RNAscope in particular in OPCs in brain sections. Our findings imply that, during normal development, OPCs thus display properties similar to that ascribed to adult OPCs and mature oligodendrocytes, which could shape the inflammatory environment, or perform a trophic role on their environment at defined time-windows.

In line with the expression of genes of the immune/inflammatory pathways in O4+ cells, both under unstressed and stressed conditions, we found that, specific binding sites for TFs involved in the immune/inflammatory pathways (members of the IRF, NFκB, and AP-1/Jun families) are occupied before and after exposure to neuroinflammation. Interestingly, members of these TF families are also at play in OPCs in the adult brain in a murine multiple sclerosis model, reinforcing our data [31]. In addition, we identified existing paired binding motifs, for these TFs, in ATAC-Seq peaks, which suggest that these TFs can act in a combinatorial mode. Interestingly, changes in their combination (and/or activity) are observed in other contexts [34] and might account for the increase in the transcription of the corresponding genes that we detect upon neuroinflammation exposure. In addition, the mRNA levels of some of these TFs show increase upon neuroinflammation, which might contribute both to changes in the combinatory arrangements of TFs of the immune/inflammatory pathways and to elevated transcription of their target genes.

Our data also nurture an underlying, emerging concept: molecules, which have been historically identified and studied as key mediators of stress responses and guardians of cell or organism homeostasis, are also pivotal in physiological conditions for normal development [35]. Emblematic examples are represented by TF families, like NFkB [36], HSFs and their target genes encoding not only the heat shock proteins, but also a large repertoire of genes crucial for development [37–40], and by critical players of the unfolded stress response (UPR; [41]). Whether these two apparently distinct functions have emerged concomitantly in evolution or not is unclear, but for technical and practical reasons, the roles of these molecules in normal development have been understudied. The stage-dependent production of inflammatory players by prenatal OPCs during the time-course of their maturation, as well as their potential role in brain formation, is therefore less unexpected than it appears. In addition to this exciting, conceptual context and to the best of our knowledge, by using unbiased approaches, we have unraveled for the first time the global physiological, constitutive expression of genes in unstressed O4+ cells, at an early murine postnatal stage, equivalent to the third trimester of pregnancy.

What could be the impact of the massive upregulation of genes of the neuroinflammatory pathway on the late OPC cell fate? Strikingly, we can reproduce the correlation between the upregulation of cytokine and chemokine gene expression and the downregulation of the myelin markers in an oligodendroglial cell line upon proinflammatory stimulus, which suggests that some causality might exist between the abnormal upregulation of immune/inflammatory modulators and the compromised myelination program in O4+ cells. In that vein, Moyon et al. [42] have pointed out the role of IL1B and CCL2 production by premyelinating OPCs in modulating their motility capacities and eventually differentiation. In addition, CXCR2 counteracts adult OPC differentiation and myelination potential, in a model of multiple sclerosis, by interfering with the PI3K/AKT/mTOR pathway, thereby strongly reinforcing the possibility of a role of cytokines and chemokines in OPC maturation blockade, during development [43]. Another possibility is that the counteracting effects of cytokines and chemokines on OPC differentiation would operate through the recruitment by OPCs of other cell types that are known to influence OPC maturation (like microglia; 44, 45). In addition, in favor of a link between expression of genes of the immune/inflammatory pathways in early postnatal late OPCs and myelination issue, we also observe overexpression of PSMB8 and PSMB9 in late OPCs upon neuroinflammation (FC at P5 = 3,153; *p* value = 2,44E-04; FC at P10 = 3,984, *p* value = 1,33E-03, respectively). PSMB8 and PSMB9 are two proteasome subunits, which belong to the immune-proteasome and are found specifically upregulated in adult OPCs, in areas of compromised remyelination, in people with longstanding multiple sclerosis, which is also coupled to inability of adult OPCs to differentiate into mature oligodendrocytes in an inflammatory demyelinating mouse model [28]. The link between exacerbation of classically immune-attributed gene expression profiles and failure to myelinate could very likely be also functional in murine early postnatal OPCs, at a period equivalent to the prenatal third trimester of pregnancy.

We show that, among the genes belonging to the immune/inflammatory pathways and undergoing dysregulated expression upon exposure to neuroinflammation in O4+ cells, only a limited number exhibit significant chromatin remodeling. Hypoxia-Inducible Factor 3, *Hif3a*, is one of them and was previously shown to be regulated, in an oxygen-independent manner, in two distinct models of inflammation, in non-neural cells [46, 47]. Interestingly, in parallel of our data, Cuomo et al. [47] established that proinflammatory cytokines are responsible for the activation of *Hif3a* gene, through epigenetic changes and the involvement of NFκB. In addition, the chromatin remodeling and upregulation of *Hif3a*, could possibly be linked to the downregulation of genes involved in paracrine ability of OPCs to promote angiogenesis and vascularization (C4 cluster), which therefore ensures energy supply for the production of myelin segments and extension of OPC plasma membrane surface *via* their encoding HIF1/2α signaling [26]. Indeed, as HIF3α lacks the transactivation domain present in the other members of the HIF family, it is thought that HIF3α acts as a negative regulator of HIF1/2α, and could therefore participate in compromising the ability of O4+ cells to promote angiogenesis and thereby contribute to myelination defects [26].

The second prominent impact of neuroinflammation is the abnormal, sustained overexpression of numerous cell cycle genes in O4+ cells at P10 (cluster C2). Such feature is surprising, since O4+ cells are postmitotic [12]. Our previous in vivo studies have established that neuroinflammation does not lead to cell proliferation, nor apoptosis in the white matter [8]. Nevertheless, the overexpression of cell cycle genes might have nevertheless important impact on O4+ cell fate. Indeed, some *Cdk* genes perform known roles in differentiation. This includes the *Cdk6* gene, whose downregulation is physiologically observed between OPCs and immOLs [12] and which we found upregulated by neuroinflammation in O4+ cells at P5 in our microarray data (FC 1.9, *p* value, 6,34E−11) as well as in the Oli-Neu cell line treated by a proinflammatory molecule (Fig. 6C). Although CDK6 has long been envisioned as a mitogenic factor, it was demonstrated that it inhibits differentiation in a diversity of cell types including oligodendrocytes [48, 49]. Therefore, the abnormal upregulation of the *Cdk6* gene in response to neuroinflammation could contribute to OPC maturation blockade.

In addition, there might be a link between the reactivation of *Cdk* genes and the activation of genes of the immune/inflammatory pathways in late OPCs. CDK6 interacts with p65, a NFκB subunit, and is necessary for its recruitment to NFκB gene targets and inflammatory genes [50]. Therefore, the upregulation of the *Cdk6* gene in response to neuroinflammation could be at least partially responsible of the activation of genes of the immune/inflammatory pathways in O4+ cells. Notably, *Cdk9* gene expression is also upregulated in O4+ cells at P5, in our microarray data (FC, 1.38; *p* value, 2,06E−14; FC 1.24; *p* value, 2,84E−12), and *Cdk9* expression is also required for inflammatory gene expression (reviewed in [51]). The dysregulation of *Cdk* genes in neuroinflammation-exposed late OPCs might thus have multiple impacts on the O4+ cell fate.

In conclusion, neuroinflammation has three major entry routes by which it perturbs the transcriptome of cell populations corresponding to late OPCs and immOLs in a broad manner, and contributes to the emergence of cell fate issues which underlie their maturation blockade: (1) massive upregulation of the innate immune/inflammatory pathways at P5, which takes advantage of a primed epigenetic landscape; (2) persistence of the cell cycle pathway at P10; and (3) the downregulation of the ability to produce pro-angiogenic molecules and components necessary for the extension of the plasma membrane — all three having potential impact on the myelination program. These genome-wide perturbations induce the emergence of intermediate “frozen” cell identity in O4+ cells, which signs OPC maturation blockade and reveal its underlying mechanisms.

Our results have important therapeutic consequences in the context of a chronic perinatal systemic inflammation: because of the striking intertwining between the injurious and developmental facets of the inflammatory modulators, whose physiological expression by OPCs is disturbed by neuroinflammation, we should reconsider the proposal that global targeting of the immune/inflammatory pathway might constitute a therapeutic option.

## Materials and methods

### Animal model

Experimental protocols were approved by the institutional review committee (under the following reference by the French Ministère de l’Enseignement Supérieur et de la Recherche (#2016040414515579) and met the guidelines for the United States Public Health Service’s Policy on Humane Care and Use of Laboratory Animals (NIH, Bethesda, MD, USA). Sex was determined at birth, and confirmed by abdominal examination at sacrifice. This animal model is similar to the human in that males are more affected and, as such, only male OF1 pups were used, since, in humans, female OPC maturation is mostly unaltered [7]. IL1B injections were performed as described [8, 9]. Five µL volume of phosphate-buffered saline (PBS) containing 10 µG/kG/injection of recombinant mouse IL1B (R&D Systems, Minneapolis, MN) or of PBS alone (control) was injected intraperitoneally (i.p.) twice a day on days P1 to P4 and once a day, on day P5 (see Fig. 1). Pups were sacrificed four hours after the morning injection of IL1B at P3 or P5, and at a similar time at P9, or P10. ATAC-Seq data were produced from 3 independent biological replicates for each condition (PBS or IL1B). Microarray data were produced from 6 independent biological replicates for each condition (PBS or IL1B), using the same animals that were also analyzed for CD11B+ microarrays [9].

### O4+ and CD11B+ microglial magnetic-activated cell sorting in mouse

O4+ cells were isolated at P3, P5, P9, or P10 by Magnetic-Activated Cell Sorting (MACS, Miltenyi Biotec, Bergisch Gladbach, Germany), according to the manufacturer’s protocol and as previously described [52]. Briefly, brains were collected without cerebellum and olfactory bulbs, pooled (3 brains per sample) and dissociated using the Neural Tissue Dissociation Kit containing papain. O4+ cells were then enriched by MACS, using the anti-O4 MicroBeads. For microarray and RT-qPCR analysis, the eluted isolated cells were centrifuged for 5 min at 600 × *g* and conserved at −80 °C. CD11B+ microglial cells were isolated as described [9]. The unlabeled fraction mainly contained astrocytes (see Fig. S2D). For the ATAC-seq experiment, 50,000 cells were immediately lysed and their nuclei submitted to Tn5 activity. The purity of the eluted O4-positive fraction was verified using RT-qPCR for Myelin Basic Protein (*Mbp*), ionizing calcium binding adapter protein (*Iba1*), glial fibrillary acid protein (*Gfap*) and neuronal nuclear antigen mRNAs (*NeuN*; Fig. S2A). Comparable numbers of O4+ cells from control (PBS) and treated (IL1B) samples were collected (1.12 × 10^6^ ± 0.12 × 10^6^ cells per sample).

### OPC culture and differentiation

OPCs were prepared from newborn OF1 mice as described [53, 54]. In brief, forebrain cortices were removed from postnatal day 0–2 mouse pups and freed from meninges. Minced tissues were enzymatically digested with 0.125% trypsin (Sigma) and 0.0025% DNase I (Sigma) for 15 min at 37 °C and then mechanically dissociated. Cells were filtered through a 100-μm-pore-size cell strainer (BD), centrifuged 10 min at 1800 rpm, resuspended in minimum essential Eagle’s medium (Sigma) supplemented with 10% FBS (Gibco), 1% Glutamax (Gibco), 1% penicillin-streptomycin (P/S) solution (Sigma), and 0.5% glucose and plated in T75 flasks at a density of 2 × 10^5^/cm^2^. Mixed glial cell cultures were grown until confluence for 9–11 days (medium was replaced every 48–72 h) and shaken for 1.5 h at 260 rpm to detach microglia. These detached microglia were then collected and removed together with the media. Remaining cells were shaken for additional 18 h to detach the OPCs from the astrocyte base layer, and were simultaneously treated with 100 µg/ml liposomal clodrosome suspension (Clodrosome®, Encapsula Nanosciences, Brentwood, USA) which selectively eliminates any residual microglia. The detached OPC cell suspension was filtered through a 20-µM-pore-size filter (Millipore) and incubated in an untreated Petri dish for 10 min at 37 °C to allow attachment of any remaining microglia. Purified OPCs were then seeded onto poly-D-lysine-coated 12-multiwell plates at a density of 3 × 10^4^/cm^2^ in OPC proliferation medium composed of Neurobasal medium (Gibco), 2% B21 (Miltenyi biotec), 1% P/S (Sigma) and 1% Glutamax (Gibco), supplemented with growth factors consisting in 10nG/mL FGFα (Sigma) and 10nG/mL PDGFα (Sigma). After 72 h, OPC differentiation was initiated by growth factor withdrawal and addition of 40 nG/mL of T3 (Sigma). At the same time, OPCs were treated with 50 nG/mL IL1B (R&D Systems, Minneapolis, MN) or PBS for 4 h, treatment was removed, new media provided and cells were grown in differentiation medium until 72 h (Fig. 4B).

### Oli-neu cell line culture and differentiation

The immortalized murine OPC cell line, Oli-neu, was kindly provided by Dr Sheila Harroch (Pasteur Institute, Paris, France). Oli-neu was established from OPC-enriched murine primary cultures from E16 brains transformed with a provirus containing the oncogene *T-Neu* [55]. Various differentiation protocols have been established, among which treatment with PD174265, a selective inhibitor of the activity of Epidermal Growth Factor receptor (ErbB) tyrosine kinase, has been shown to induce MBP expression [56]. These cells were cultured in Dulbecco’s modified Eagle’s minimum essential medium (DMEM) containing Glutamax 1× and high glucose (4.5 G/L; Gibco 31966), supplemented with 1 mG/mL insulin (Sigma), N2 supplement (Gibco), 100 μG/mL T4 and T3 (Sigma), 1% horse serum (Gibco), and 1% P/S (Sigma). At confluence, the cells were mechanically detached and seeded in 12-multiwell plates at a density of 3 × 10^4^ cells/cm^2^. After 24 h, differentiation was induced by addition of 1 µM PD174265 (ChemCruz) diluted in DMSO at 1 mM or 1:1 volume of neuron-conditioned medium obtained as described in [57]. Medium was replaced after 48 h and differentiation was stopped after 72 h (Fig. S1E; Fig. 4C; Fig. 6C). Inflammatory stimulus was provided by exposing Oli-neu cells to 10 ng/ml TNFα (Tumor necrosis factor α).

### RT-qPCR analysis and Luminex assay

Preparation of samples for quantitative reverse-transcriptase polymerase-chain reaction (RT-qPCR), primer design PCR protocol and Luminex assay were similar to that previously described ([58]; Bio-Plex Pro Mouse Cytokine 23-Plex Assay kit, #M60009RDPD; Biorad, France). Primer sequences are given in Table S13. *Gapdh* (glyceraldehyde-3-phosphate dehydrogenase gene) and *Rpl13* (Ribosomal Protein L13) were chosen to standardize the quantitative experiments based on reference gene suitability testing.

### ATAC-Seq analysis in O4+ cells

ATAC-seq protocol was performed as described [15] with slight modifications. In brief, cells were immediately lysed after cell sorting and a total of 50,000 nuclei were subjected to Tn5-mediated transposition for 30 min, resulting in ‘tagmented’ DNA fragments. Tagmented DNA was purified on MinElute colums (Qiagen) and amplified/tagged in two steps using NEBnext High-Fidelity 2× PCR master mix (New England Biolabs). Amplified DNA was purified twice with 1.8 volumes of NucleoMag NGS Clean-up and Size Select beads (Macherey Nagel). DNA was quantified using the Qubit dsDNA HS Assay Kit and the quality of each library determined on Agilent 2100 Bioanalyzer DNA High Sensitivity ChIPs. Libraries demonstrating appropriate nucleosomal profiles were multiplexed and subjected to Illumina NextSeq500 sequencing (IGenSeq Platform, ICM, Paris, France). The main steps of sequence analyses are summarized in Fig. S3A and detailed on github (https://github.com/parisepigenetics/ATAC-seq/). After quality controls (Fastqc and Trimmomatic 0.33), reads were aligned on the mm10 genome with Bowtie 2 (Galaxy tool version 2.3.4.1 ([59]; default parameters; Table S5; Fig. S3A). Peak calling was performed with MACS2.2.0; default parameters; *q* < 0.05) separately for the two conditions, using a pooled (*n* = 3) bam file of control samples and a pooled (*n* = 3) bam file of IL1B samples. The two resulting bed files were merged and, after removing the mm10 blacklist (http://mitra.stanford.edu/kundaje/akundaje/release/blacklists/mm10-mouse/mm10.blacklist.bed.gz), 213,246 DNA regions (peaks) significantly detected in at least one condition were delimitated (Table S5). The number of reads was determined in each peak for each sample using Bedtools coverage (version 2.19.1) and normalized to the library sizes. Principal component analysis (PCA) was performed on log transformed read count values of the top 500 most variable peaks, using the prcomp function in R. Differential peak detection between the three PBS and the three IL1B samples was performed with the Bioconductor software package EdgeR (3.22.3; [17]), using R studio (0.98.1103; http://www.rstudio.com). Statistical comparison was performed using the exact test function followed by False Discovery Rate (FDR) determination by the Benjamini-Hochberg method. Raw data are available under SRA BIOPROJECT accession # PRJNA540409.

### Linking of HAEC and O4+ cell ATAC-Seq datasets

We used a public ATAC-Seq dataset of human aortic endothelial cells (HAECs [19]; NCBI Gene Expression Omnibus; accession no: GSE89970) and processed the raw reads (using the hg19 reference genome) to obtain a set of peaks. Both sets of peaks (control and IL1B-treated samples) were annotated using HOMER’s annotatePeaks function. Next, HAEC peaks were matched to mouse O4+ cell peaks through gene annotations, by taking only those peaks annotated with matching orthologous genes (only 1-to-1 orthology was considered). Matching was further restricted to promoter regions (peaks with a relative maximum distance of 2 kb from the TSS). In order to ensure that peaks were true matches, this set was further restricted to a relative distance of 500 bp from each other in relation to the TSS. Using this approach, a total of 7739 peaks were matched between the HAEC and O4+ cell datasets, including 100 peaks identified as differential in the HAEC dataset using DESeq2 (Table S8). Next, the number of reads mapped to matched peaks were obtained by counting the number of reads at the summit ± 50 bp using the featureCounts package of the Subread software (v1.6.0) and the counts were normalized against the total number of reads present in all matched peaks and converted into reads per million. Normalized read number distributions of the two datasets were compared using the one-sample Wilcoxon rank test with continuity correction.

### Microarrays of mouse O4+ cell gene expression and data preprocessing

Microarray analysis was performed on six control and six IL1B samples (O4+ cells isolated at P5 or P10 after in vivo PBS or IL1B treatment) using Agilent Whole Mouse Genome Oligo Microarrays 8 × 60K (Agilent). Raw data are available in NCBI Gene Expression Omnibus; accession no: GEO GSE197563. All the steps, from RNA extraction to statistical analysis, were performed by Miltenyi Biotec, as previously described [53]. In brief, intensity data were subjected to quantile normalization, unpaired t-tests (equal variance) were conducted to compare intensities between the two groups for each probe and p-values were adjusted through FDR determination by the Benjamini-Hochberg method. Fold changes correspond to the median ratios (median [IL1B]/median [PBS]). When FC < 1, the FC was expressed as a negative value using the formula FC(neg)=-1/FC. For example, if FC = 0.5, the indicated FC is −2. Probes with FDR < 0.05 were considered significant. An additional fold change (FC) threshold was chosen at ±1.5 (corresponding to FC > 1.5 and <0.666).

### Heat map representation

Heat maps were created using Morpheus (https://software.broadinstitute.org/morpheus). The Log2 median-centered data were visualized using a fixed (nonrelative) color pattern. The color scales are indicated on each heat map. Rows and columns were submitted to hierarchical clustering with the following criteria: metric = one minus Pearson correlation, linkage method = average.

### GO-term enrichment analysis

GO-term Biological Pathway enrichment was done using David 6.8[60, 61].

### TFBS motif enrichment analysis, and TF footprint analysis

The 213,246 significant peaks detected by MACS2 in at least one condition (PBS or IL1B) were annotated with the *HOMER annotatePeaks* function. The list was restricted to the peaks located between −8 kb and +8 kb from the closest TSS (“TSS-All” list). Among this list, peaks were selected, which were annotated with a gene name and for which the gene expression was modulated in the microarray analysis (FDR < 0.05 and FC > 1.5 or < -1.5). The full list of peaks and lists restricted to up or downregulated genes were submitted to motif enrichment analysis using *HOMER FindMotifsGenome* with the options “-size given” and “-mask”. The “TSS-All” list was used as background. Six motifs were then localized in the full list of peaks (ALL) with *HOMER FindMotifs*. The six chosen motifs corresponded to the top 5 motifs enriched in the full list of peaks (ISRE, IRF1, IRF2, Nfkb-p65 and PGR, Fig. S5B) and the 15th motif enriched in the list restricted to upregulated genes (PU1:IRF8, Fig. S5A). Indeed, this last motif was the most significantly enriched motif belonging to the list of paired motifs (Fig. 7A). For each of these motifs, the average profile of Tn5 activity was visualized using *pyDNAse* dnase_average_profile.py; [21]. This profiling was performed using a pooled bam file of PBS samples, or a pooled bam file of IL1B samples separately, and a pooled bam file of the two conditions (“both”) together.

### Testing for enrichment of paired motifs (PMET)

2319 ATAC-Seq peaks corresponding to 1266 upregulated genes (886 different gene names) and 946 ATAC-seq peaks corresponding to 454 downregulated (336 different gene names) were tested for significantly enriched pairs of TFBS relative to a universe containing all the peaks located ±8 kb around the closest TSS. For each individual motif from the homer database, all peaks in the universe were ranked by motif occupancy using a binomial score. Then for every possible pair of motifs, peaks containing both motifs were identified using the overlap between top 5000 ranked peaks for each of the individual motifs. A hypergeometric test was used to calculate the enrichment score (p-value) for the overlap between each test set and the peaks containing both motifs. The resulting *p* values were corrected using the Benjamini-Hochberg correction. The PMET source code is available on GitHub at: https://github.com/kate-wa/PMET-software.

### Single-cell analysis of the Jin et al. [14] data

Single-cell RNA-seq data by Jin et al. [14] were analyzed using the Seurat R package v.4.1.0 [62]. Unsupervised clustering was performed and marker genes (*Pdgfra*, *Olig2*, *Sox10*, *Gpr17*) were used to identify the OPC cluster. Seurat’s AddModuleScore function was used to calculate the module scores (the average expression of the immune/inflammation gene set subtracted by the aggregated expression of randomly selected control feature sets (Table S3 and Fig. 3B-D). RMarkDown is provided in the Supplementary Files.

### Statistical analysis

All in vivo and in vitro experiments were performed using an alternating treatment allocation. All analyses were performed by an experimenter blinded to the treatment groups. The results of qRT-PCR and Luminex analyses are expressed as mean ± SEM of at least four independent experiments; the number of analyzed samples is indicated in the figure legends or on the graphs. Statistical analysis was done using the nonparametric Mann-Whitney t-test with Graphpad 5.0 software (San Diego, CA, USA) or two-way ANOVA followed by Bonferroni - Post Hoc Test as indicated in each figure legend. Significance is shown on the graphs (*p < 0.05; **p < 0.01; ***p < 0.001). Specific statistical analyses for ATAC-seq and microarray analyses are detailed in the dedicated sections of Material and Methods. The significance of intersection between the two datasets was evaluated by hypergeometric test (Phyper function) in R studio.

## Supplementary information

Supplementary Figures

Table S1

Table S2

Table S3

Table S4

Table S5

Table S6

Table S7

Table S8

Table S9

Table S10

Table S11

Table S12

Table S13

ATAC-seq workflow

RMarkDown workflow for scAnalysis fig3&S3

## Data Availability

All datasets generated and analyzed during this study are included in this published article and its Supplementary information files. Additional data are available from the corresponding author on reasonable request.

## References

[1] Delobel-Ayoub M, Arnaud C, White-Koning M, Casper C, Pierrat V, Garel M (2009). Behavioral problems and cognitive performance at 5 years of age after very preterm birth: the EPIPAGE study. Pediatrics.

[2] Volpe JJ (2009). Brain injury in premature infants: a complex amalgam of destructive and developmental disturbances. Lancet Neurol.

[3] Bokobza C, Van Steenwinckel J, Mani S, Mezger V, Fleiss B, Gressens P (2019). Neuroinflammation in preterm babies and autism spectrum disorders. Pediatr Res.

[4] Ball G, Boardman JP, Rueckert D, Aljabar P, Arichi T, Merchant N (2012). The effect of preterm birth on thalamic and cortical development. Cerebral Cortex.

[5] Billiards SS, Haynes RL, Folkerth RD, Borenstein NS, Trachtenberg FL, Rowitch DH (2008). RESEARCH ARTICLE: myelin abnormalities without oligodendrocyte loss in periventricular leukomalacia: white matter analysis in PVL. Brain Pathology.

[6] Leviton A, Gressens P (2007). Neuronal damage accompanies perinatal white-matter damage. Trends in Neurosciences.

[7] Hagberg H, Mallard C, Ferriero DM, Vannucci SJ, Levison SW, Vexler ZS (2015). The role of inflammation in perinatal brain injury. Nat Rev Neurol.

[8] Favrais G, van de Looij Y, Fleiss B, Ramanantsoa N, Bonnin P, Stoltenburg-Didinger G (2011). Systemic inflammation disrupts the developmental program of white matter. Ann Neurol.

[9] Krishnan ML, Van Steenwinckel J, Schang AL, Yan J, Arnadottir J, Le Charpentier T (2017). Integrative genomics of microglia implicates DLG4 (PSD95) in the white matter development of preterm infants. Nat Commun.

[10] Shiow LR, Favrais G, Schirmer L, Schang AL, Cipriani S, Andres C (2017). Reactive astrocyte COX2-PGE2 production inhibits oligodendrocyte maturation in neonatal white matter injury. Glia.

[11] Rangon CM, Schang AL, Van Steenwinckel J, Schwendimann L, Lebon S, Fu T (2018). Myelination induction by a histamine H3 receptor antagonist in a mouse model of preterm white matter injury. Brain Behavior Immun.

[12] Boccazzi M, Van Steenwinckel J, Schang AL, Faivre V, Le Charpentier T, Bokobza C (2021). The immune-inflammatory response of oligodendrocytes in a murine model of preterm white matter injury: the role of TLR3 activation. Cell Death Dis.

[13] Zhang Y, Chen K, Sloan SA, Bennett ML, Scholze AR, O’Keeffe S (2014). An RNA-sequencing transcriptome and splicing database of glia, neurons, and vascular cells of the cerebral cortex. J Neurosci.

[14] Jin X, Simmons SK, Guo A, Shetty AS, Ko M, Nguyen L (2020). In vivo Perturb-Seq reveals neuronal and glial abnormalities associated with autism risk genes. Science.

[15] Buenrostro JD, Giresi PG, Zaba LC, Chang HY, Greenleaf WJ (2013). Transposition of native chromatin for fast and sensitive epigenomic profiling of open chromatin, DNA-binding proteins and nucleosome position. Nat Methods.

[16] Zhang Y, Liu T, Meyer CA, Eeckhoute J, Johnson DS, Bernstein BE (2008). Model-based analysis of ChIP-Seq (MACS). Genome Biol.

[17] Robinson MD, McCarthy DJ, Smyth GK (2010). edgeR: a Bioconductor package for differential expression analysis of digital gene expression data. Bioinformatics.

[18] Buenrostro JD, Wu B, Chang HY, Greenleaf WJ (2015). ATAC-seq: A method for assaying chromatin accessibility genome-wide. Curr Protoc Mol Biol.

[19] Hogan NT, Whalen MB, Stolze LK, Hadeli NK, Lam MT, Springstead JR (2017). Transcriptional networks specifying homeostatic and inflammatory programs of gene expression in human aortic endothelial cells. eLife.

[20] Yanai H, Negishi H, Taniguchi T (2012). The IRF family of transcription factors: Inception, impact and implications in oncogenesis. OncoImmunology.

[21] Piper J, Elze MC, Cauchy P, Cockerill PN, Bonifer C, Ott S (2013). Wellington: a novel method for the accurate identification of digital genomic footprints from DNase-seq data. Nucleic Acids Res.

[22] Green B, Bouchier C, Fairhead C, Craig NL, Cormack BP (2012). Insertion site preference of Mu, Tn5, and Tn7 transposons. Mobile DNA.

[23] Fu MM, McAlear TS, Nguyen H, Oses-Prieto JA, Valenzuela A, Shi RD (2019). The Golgi outpost protein TPPP nucleates microtubules and is critical for myelination. Cell.

[24] Snaidero N, Möbius W, Czopka T, Hekking LHP, Mathisen C, Verkleij D (2014). Myelin membrane wrapping of CNS axons by PI(3,4,5)P3-dependent polarized growth at the inner tongue. Cell.

[25] Baron W, Hoekstra D (2010). On the biogenesis of myelin membranes: sorting, trafficking and cell polarity. FEBS Letters.

[26] Yuen TJ, Silbereis JC, Griveau A, Chang SM, Daneman R, Fancy SPJ (2014). Oligodendrocyte-encoded HIF function couples postnatal myelination and white matter angiogenesis. Cell.

[27] Chavali M, Ulloa-Navas MJ, Pérez-Borredá P, Garcia-Verdugo JM, McQuillen PS, Huang EJ (2020). Wnt-dependent oligodendroglial-endothelial interactions regulate white matter vascularization and attenuate injury. Neuron.

[28] Kirby L, Jin J, Cardona JG, Smith MD, Martin KA, Wang J (2019). Oligodendrocyte precursor cells present antigen and are cytotoxic targets in inflammatory demyelination. Nat Commun.

[29] Vihervaara A, Mahat DB, Guertin MJ, Chu T, Danko CG, Lis JT (2017). Transcriptional response to stress is pre-wired by promoter and enhancer architecture. Nat Commun.

[30] Vihervaara A, Duarte FM, Lis JT (2018). Molecular mechanisms driving transcriptional stress responses. Nat Rev Genet.

[31] Meijer M, Agirre E, Kabbe M, van Tuijn CA, Heskol A, Zheng C (2022). Epigenomic priming of immune genes implicates oligodendroglia in multiple sclerosis susceptibility. Neuron.

[32] Zeis T, Enz L, Schaeren-Wiemers N (2016). The immunomodulatory oligodendrocyte. Brain Res.

[33] Cahoy JD, Emery B, Kaushal A, Foo LC, Zamanian JL, Christopherson KS (2008). A transcriptome database for astrocytes, neurons, and oligodendrocytes: a new resource for understanding brain development and function. J Neurosci.

[34] Mancino A, Termanini A, Barozzi I, Ghisletti S, Ostuni R, Prosperini E (2015). A dual cis-regulatory code links IRF8 to constitutive and inducible gene expression in macrophages. Genes Dev.

[35] Cardona AE, Li M, Liu L, Savarin C, Ransohoff RM (2008). Chemokines in and out of the central nervous system: much more than chemotaxis and inflammation. J Leukoc Biol.

[36] Espín-Palazón R, Traver D (2016). The NF-κB family: key players during embryonic development and HSC emergence. Exp Hematol.

[37] Chang Y, Ostling P, Akerfelt M, Trouillet D, Rallu M, Gitton Y (2006). Role of heat-shock factor 2 in cerebral cortex formation and as a regulator of p35 expression. Genes Dev.

[38] El Fatimy R, Miozzo F, Le Mouël A, Abane R, Schwendimann L, Sabéran-Djoneidi D (2014). Heat shock factor 2 is a stress-responsive mediator of neuronal migration defects in models of fetal alcohol syndrome. EMBO Mol Med.

[39] Gomez-Pastor R, Burchfiel ET, Thiele DJ (2018). Regulation of heat shock transcription factors and their roles in physiology and disease. Nat Rev Mol Cell Biol.

[40] Duchateau A, de Thonel A, El Fatimy R, Dubreuil V, Mezger V (2020). The « HSF connection »: Pleiotropic regulation and activities of Heat Shock Factors shape pathophysiological brain development. Neurosci Lett.

[41] Laguesse S, Creppe C, Nedialkova DD, Prévot PP, Borgs L, Huysseune S (2015). A dynamic unfolded protein response contributes to the control of cortical neurogenesis. Dev Cell.

[42] Moyon S, Dubessy AL, Aigrot MS, Trotter M, Huang JK, Dauphinot L (2015). Demyelination causes adult CNS progenitors to revert to an immature state and express immune cues that support their migration. J Neurosci.

[43] Wang L, Yang H, Zang C, Dong Y, Shang J, Chen J (2020). CXCR2 antagonism promotes oligodendrocyte precursor cell differentiation and enhances remyelination in a mouse model of multiple sclerosis. Neurobiol Dis.

[44] Jana M, Pahan K (2005). Redox regulation of cytokine-mediated inhibition of myelin gene expression in human primary oligodendrocytes. Free Radic Biol Med.

[45] Balabanov R, Strand K, Goswami R, McMahon E, Begolka W, Miller SD (2007). Interferon-gamma-oligodendrocyte interactions in the regulation of experimental autoimmune encephalomyelitis. J Neurosci.

[46] Kumar H, Lim JH, Kim IS, Choi DK (2015). Differential regulation of HIF-3α in LPS-induced BV-2 microglial cells: Comparison and characterization with HIF-1α. Brain Res.

[47] Cuomo F, Coppola A, Botti C, Maione C, Forte A, Scisciola L (2018). Pro-inflammatory cytokines activate hypoxia-inducible factor 3α via epigenetic changes in mesenchymal stromal/stem cells. Sci Rep.

[48] Grossel MJ, Hinds PW (2006). Beyond the cell cycle: a new role for Cdk6 in differentiation. J Cell Biochem.

[49] Grossel MJ, Hinds PW (2006). From cell cycle to differentiation: an expanding role for cdk6. Cell Cycle.

[50] Handschick K, Beuerlein K, Jurida L, Bartkuhn M, Müller H, Soelch J (2014). Cyclin-dependent kinase 6 is a chromatin-bound cofactor for NF-κB-dependent gene expression. Mol Cell.

[51] Sundar V, Vimal S, Sai Mithlesh MS, Dutta A, Tamizhselvi R, Manickam V (2021). Transcriptional cyclin-dependent kinases as the mediators of inflammation-a review. Gene.

[52] Schang AL, Van Steenwinckel J, Chevenne D, Alkmark M, Hagberg H, Gressens P (2014). Failure of thyroid hormone treatment to prevent inflammation-induced white matter injury in the immature brain. Brain Behav Immun.

[53] McCarthy KD, de Vellis J (1980). Preparation of separate astroglial and oligodendroglial cell cultures from rat cerebral tissue. J Cell Biol.

[54] Pansiot J, Pham H, Dalous J, Chevenne D, Colella M, Schwendimann L (2016). Glial response to 17β-estradiol in neonatal rats with excitotoxic brain injury. Exp Neurol.

[55] Jung M, Krämer E, Grzenkowski M, Tang K, Blakemore W, Aguzzi A (1995). Lines of murine oligodendroglial precursor cells immortalized by an activated neu tyrosine kinase show distinct degrees of interaction with axons in vitro and in vivo. Eur J Neurosci.

[56] Gobert RP, Joubert L, Curchod ML, Salvat C, Foucault I, Jorand-Lebrun C (2009). Convergent functional genomics of oligodendrocyte differentiation identifies multiple autoinhibitory signaling circuits. Mol Cell Biol.

[57] Fratangeli A, Parmigiani E, Fumagalli M, Lecca D, Benfante R, Passafaro M (2013). The regulated expression, intracellular trafficking, and membrane recycling of the P2Y-like receptor GPR17 in Oli-neu oligodendroglial cells. J Biol Chem.

[58] Chhor V, Le Charpentier T, Lebon S, Oré MV, Celador IL, Josserand J (2013). Characterization of phenotype markers and neuronotoxic potential of polarised primary microglia in vitro. Brain Behav Immun.

[59] Afgan E, Baker D, van den Beek M, Blankenberg D, Bouvier D, Čech M (2016). The Galaxy platform for accessible, reproducible and collaborative biomedical analyses: 2016 update. Nucleic Acids Res.

[60] Huang DW, Sherman BT, Lempicki RA (2009). Systematic and integrative analysis of large gene lists using DAVID bioinformatics resources. Nat Protoc.

[61] Huang DW, Sherman BT, Lempicki RA (2009). Bioinformatics enrichment tools: paths toward the comprehensive functional analysis of large gene lists. Nucleic Acids Res.

[62] Hao Y, Hao S, Andersen-Nissen E, Mauck WM, Zheng S, Butler A (2021). Integrated analysis of multimodal single-cell data. Cell.

