# Supplementary material for: Epigenetic priming of immune/inflammatory pathways activation and abnormal activity of cell cycle pathway in a perinatal model of white matter injury
Source: Cell Death Dis. 2022 Dec 13;13(12):1038. doi: 10.1038/s41419-022-05483-4 (PMC9748018; doi:10.1038/s41419-022-05483-4)
Supplement: Supplementary file 1 — Supplementary Figures [file 41419_2022_5483_MOESM1_ESM.pdf]

# SUPPLEMENTARY INFORMATION

Schang et al.,
"Epigenetic priming of immune/inflammatory pathway activation and abnormal activity of cell cycle pathways in a perinatal model of white matter injury"

Contains:

**- Supplementary Figures S1 to S5 and their legends** (pages 2-11)

**- List and Legends of Supplementary Tables S1 to S13 and of bioinformatics workflows** (pages 12-13)

- **Supplementary References** (page 13)

Schang et al.

**Figure S1**

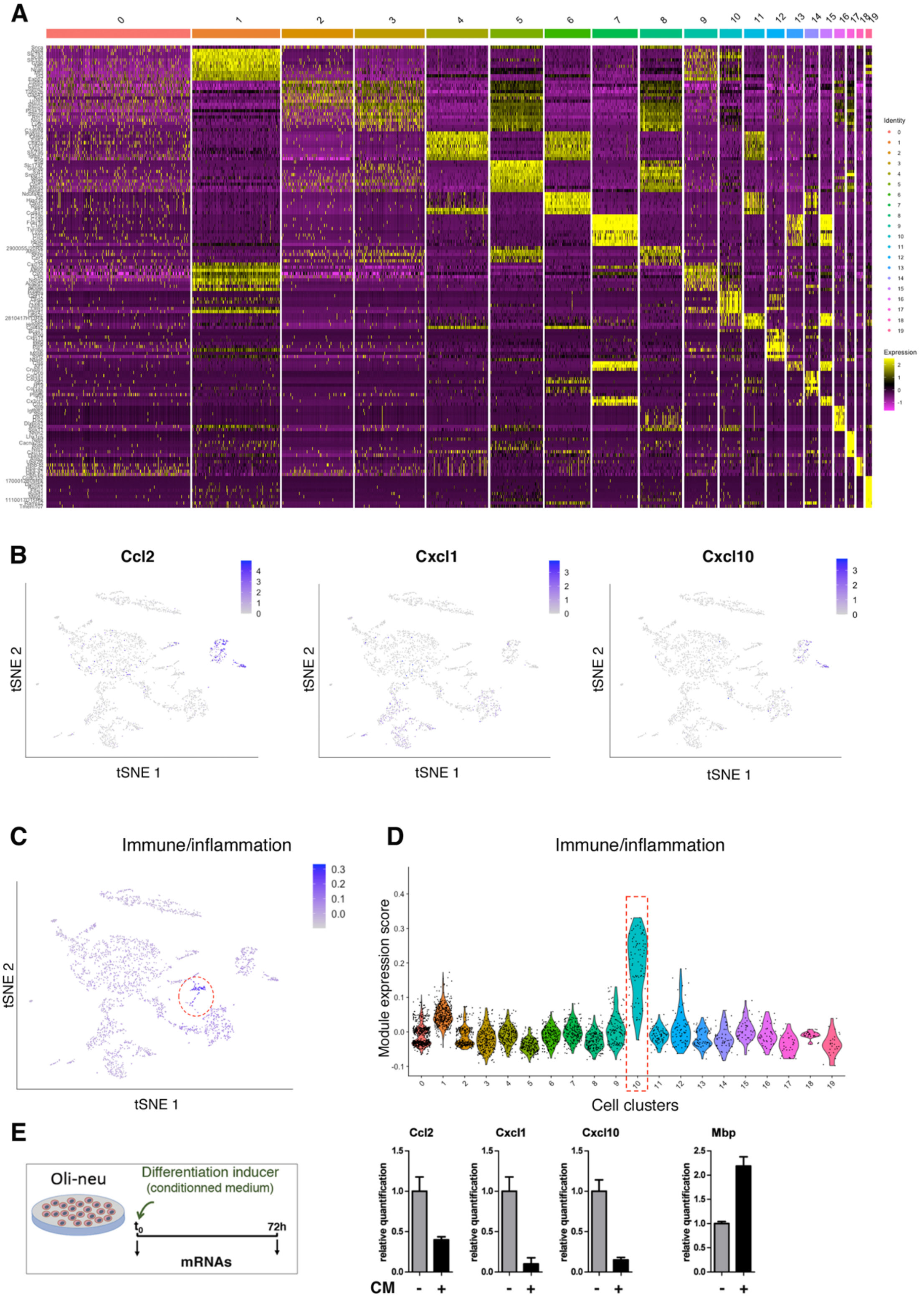


**Figure S1. Single-cell analysis of whole cortices at P7 reveals** (related to Figures 3 B-D)

(Continued next page)

**A *Heatmap of the top 10 markers (differentially expressed genes) are plotted for each cluster.*** Yellow: high expression level. Purple: low expression level. (log-normalized counts). (Cluster numbers and colors are the same as in Figure 3B).

**B *The expression of some cytokine and chemokine genes (Ccl2, Cxcl1, and Cxcl10) are already downregulated*** in P7 mouse cortices in the Pdgfra+/Olig2+ cell cluster 10, as expected from our data at P10 (Figure 2C,D).

**C**,**D** ***Genes, which belong to the TOP5 GO-terms of the gene cluster C1, show the highest expression in the Pdgfra+/Olig2+ cells that correspond to cell cluster 10***. Same **as in Figure 3** (**C,D**) but without the *Pdgfra and Gpr17* genes, markers of the oligodendrocyte precursor cells. Here, we verified that the presence of these genes in the list did not bias the result that cell cluster 10 shows the highest expression of a number of genes of the immune/inflammatory pathway listed in Figure 3C, since by removing these genes, we find the same expression profiles. Cell cluster 10 corresponds to *Pdgfra+/Olig2+ cells,* and therefore to *OPCs,* exhibits the highest gene expression levels, and is indicated by the red dotted circle in (**C**) and red rectangle in (**D**).

**E *The oligodendroglial cell line Oli-neu recapitulates the constitutive expression of cytokine and chemokine gene at the immature state and its downregulation upon differentiation observed along the maturation time-course***. RT-qPCR analyses in the Oli-neu cell line before or after 72 hours of differentiation triggered by exposure (+) to conditioned medium (CM) from primary neuron culture (see Materials and Methods and Figure 3E).

## Figure S2

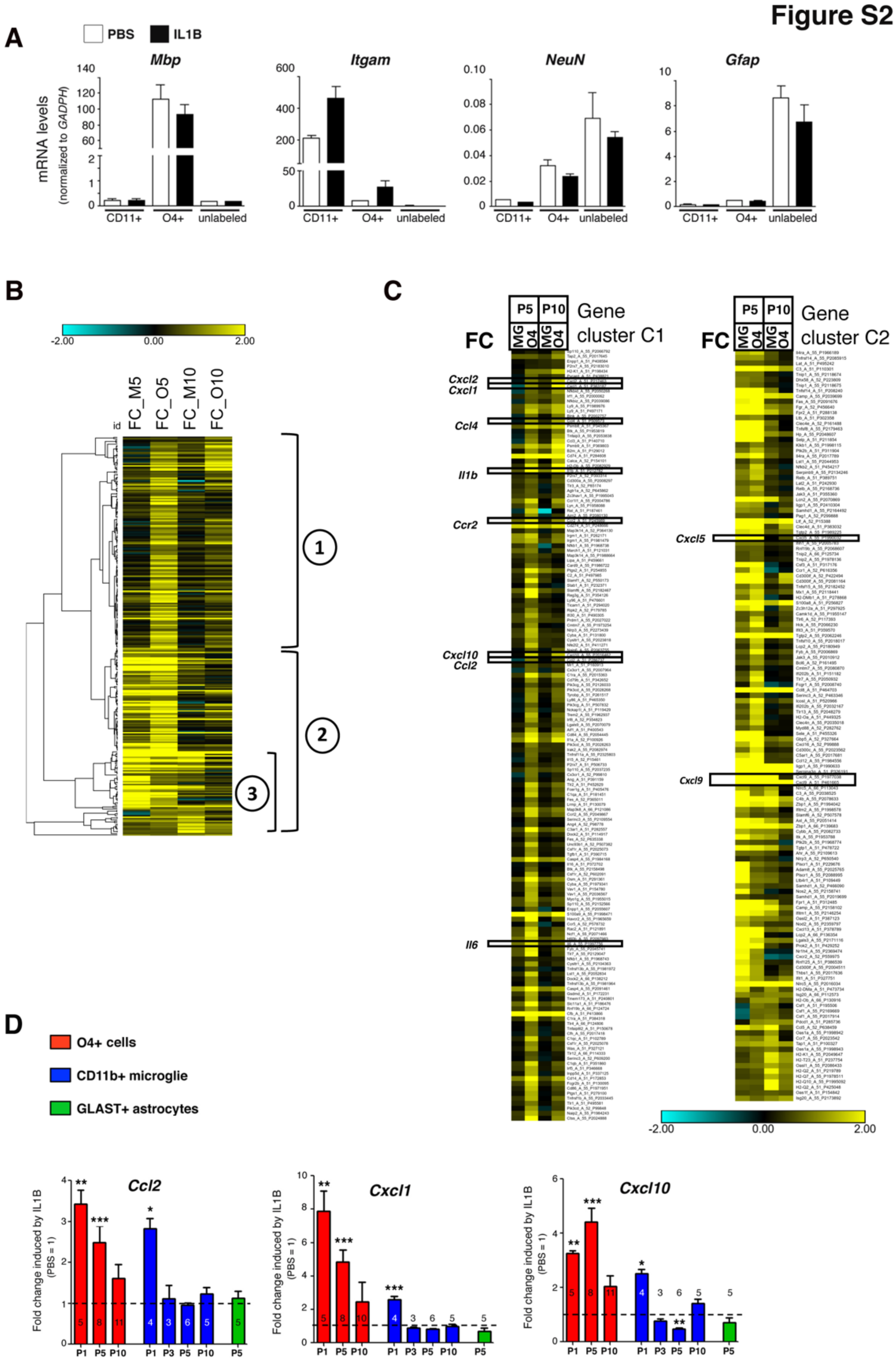


**Figure S2. Assessment of the purity of the isolated O4+ cell population** (related to Figure 4).

(Continued next page)

**Figure S2. Assessment of the purity of the isolated O4+ cell population** (related to Figure 4).

**A *Quality assessment of the O4+ cell purification process*** (relative to Figure 1) RT-qPCR experiments on the O4+ cells collected at P5, CD11B+ microglia (MG; (1); n=3 PBS samples and n=3 IL1B samples) and unlabeled cell populations, showing that, in contrast to MG (CD11B+) and astrocytes (unlabeled), the population of O4+ cells used in this study express the *Mbp* gene, whereas it exhibits very low mRNA levels of the microglia marker CD11 (encoded by the *Itgam* gene), the astrocyte marker *Gfap*, or of the neuronal marker *NeuN*. Note that *NeuN* is very lowly expressed, even in the unlabeled population, which mainly contains astrocytes, because neurons poorly survive this MACS protocol.

**B *Isolated O4+ cells and isolated CD11B+ microglial cells exhibit distinct induction profiles of immune/inflammatory gene expression in response to neuroinflammation.*** Heatmap comparison of the microarray data of the 262 upregulated genes of the immune/inflammatory pathway in isolated O4+ (top5, described in Figure 2A,B) with the corresponding genes in CD11B+ microglial cells (1). Notably, these data were produced from cells originating from the same brains, at the same timepoint (these O4+ and CD11B+ cell populations were sequentially isolated from the same animals at P5 or P10). Heatmap color scale of log2 [-2.0;+2.0]. In group 1, genes are markedly increased in O4+ cells and not MG at P5, and this profile is still observed at P10, although at a lesser extend for some probes. In group 2, genes are induced comparably in microglia and O4+ cells (and sometimes more in MG than in O4+ cells (group 3)).

**C *Distinct perturbations in gene expression profiles in isolated O4+ cells and CD11B+ MG at P5 and P10, upon neuroinflammation*.** Heat maps of the overall comparison of neuroinflammation-induced transcriptomic changes. (related to Figure 4). O4+ cells exhibit profiles of transcriptomic modifications globally very different from that of CD11B+ microglial cells (data by Krishnan et al. (1)). Examples of genes encoding cytokines and chemokines are pointed out by rectangles. Most of them are not upregulated in CD11B+ microglial cells at P5, in contrast to what happens in O4+ cells. Log2 Fold change ([-2.0; +2.0]; IL1B/PBS). M5 and M10: CD11b+ MACS- isolated microglia respectively, from P5 and P10 animals. O5: O4+ MACS isolated cells from P5 animals. O10: O4+ MACS isolated cells from P10 animals.

**D *Unique signature for inflammatory gene expression in isolated O4+ cells*, *compared to CD11B+ microglia, and GLAST+ astrocytes*.** Fold-change in the expression of genes of the immune and inflammatory pathways as detected by RT-qPCR analyses at different postnatal stages. mRNA levels are normalized to *Gapdh* for O4+ cells and astrocytes and *Rpl13* for microglia based on in-house reference gene testing. The numbers of independent experiments are indicated on each plot. *, $p < 0.05$; **, $p < 0.01$; ***, $p < 0.001$.

**Supplemental information related to Figure S2. The strong upregulation of genes belonging to the innate immune/inflammatory pathway (gene cluster C1) is not due to contamination of the O4+ isolated cell population by microglia**.

First, by performing RT-qPCR analyses, we demonstrated that these populations strongly expressed *Myelin binding protein* (*Mbp*) mRNAs, a marker of myelinating oligodendrocytes, whose transcription starts at the pre-myelinating stage, which is not the case of microglial (CD11B+) MACS-isolated fractions (Figure S2A). Conversely, the levels of the microglia marker mRNAs, CD11B (*Itgam*, *Integrin alpha M* gene) were almost undetectable in the O4+ cell population. These data show that the cell population isolated by our MACS-protocol is predominantly enriched with O4+ cells and exhibits, as expected, hallmarks of maturation arrest in response to neuroinflammation induced by intraperitoneal administration of IL1B (see also Figure 1B). Second, we ruled out that microglia (MG)

could account for the expression of genes of the immune/inflammatory pathways in our MACS-isolated O4+ cell populations. Indeed, we have previously published microarray analyses of the transcriptomic profiles in MG in this model of neuroinflammation, (CD11B+ MACS-isolated MG cells; (1); (2)). These CD11B+ cells were obtained from the same animals as the O4+ cell populations of this study, by sequential MACS-based isolation of O4+ cells and CD11B+ microglia cells. We thus compared the microarray gene expression profiles in the previously assessed CD11B+ cells to the list of 262 gene names of the C1 immune/inflammatory pathway cluster that we identified in O4+ cells (Figure 2A and Figure S2B; dataset from (1).  The gene expression profiles of O4+ cells and CD11B+ microglia populations exhibited remarkable differences in response to neuroinflammation, in terms of magnitude and direction of changes (Figure S4A,B). Notably, the upregulation of cytokine and chemokine transcripts of the C1 gene cluster was greatest at in O4+ cells than in microglia (Figure S2B,C). We confirmed these observations in independent O4+ cell and CD11B+ microglia samples, using by RT-qPCR (illustrated here for *Ccl2*, *Cxcl1* and *Cxcl10*; Figure S2D):  whereas the expression of these genes peaked at P1 in microglia, it had already recovered at P5, reaching basal levels comparable to that of PBS samples. In marked contrast, their expression in O4+ cells remained elevated ((1) ; Figure S2B, gene cluster C1). In addition, induction of cytokine and chemokine mRNAs was not observed in astrocytes at P5 under neuroinflammation conditions (GLAST+ MACS isolation; Figure S2D). In conclusion, neither CD11B+ MG, nor GLAST+ astrocytes showed any increase in the expression of selected cytokines and chemokines at P5, in contrast to O4+ cells. Our results indicate that, in our microarray analyses, the upregulation of genes of the immune/inflammatory pathways in response to neuroinflammation cannot be attributed to contamination of O4+ cells by microglia, nor astrocytes.

Schang et al.

**Figure S3**

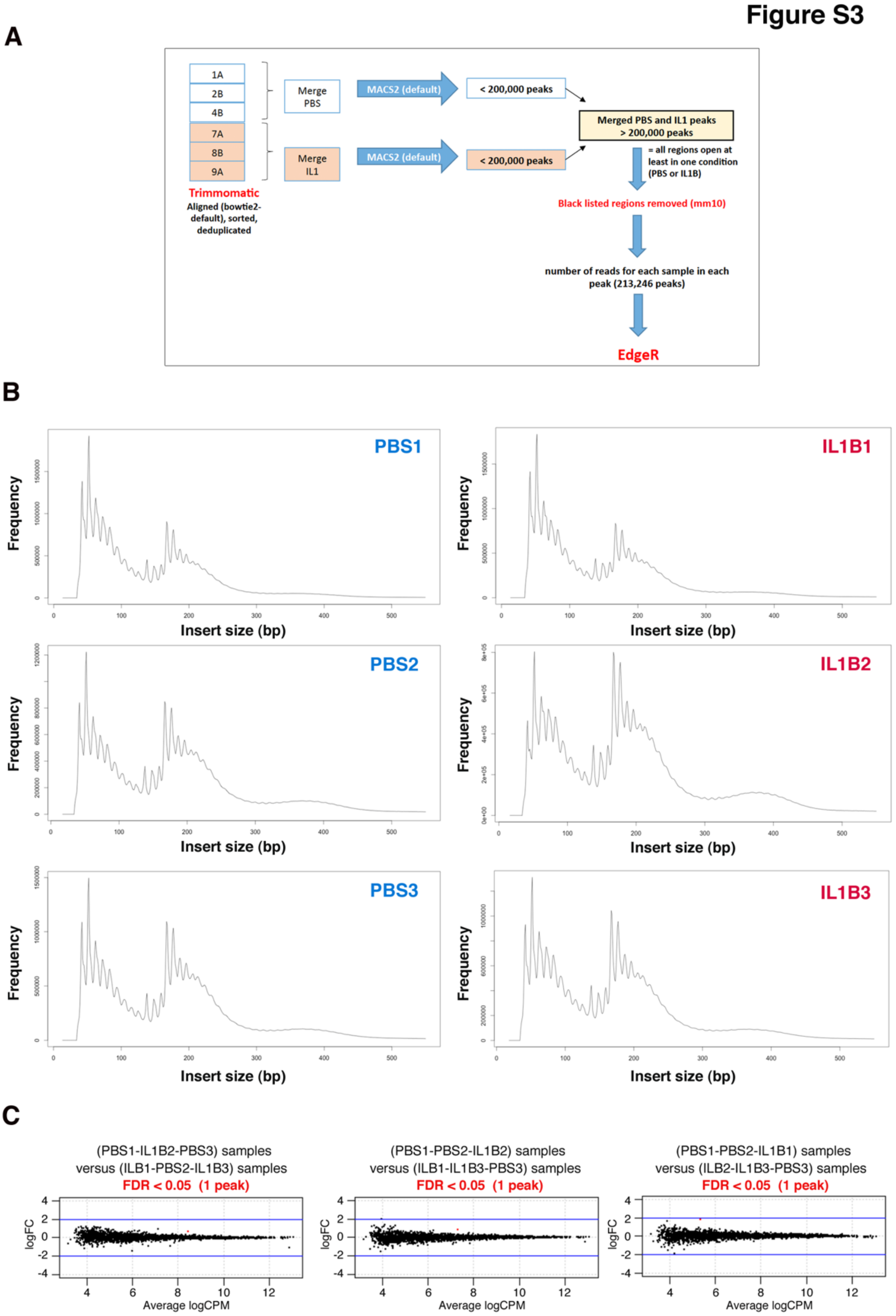


**Figure S3. ATAC-Seq data quality control.** (related to Figure 5)

(Continued next page)

**Figure S3. ATAC-Seq data quality control.** (related to Figure 5)

**A *Schematic representation of the bioinformatics and statistics workflow used for the analysis of ATAC-Seq data***

**B *Fragment-length distributions in ATAC-Seq samples***. Insert size distribution shows visible large periodicity of the nucleosome-free, mononucleosomal and dinucleosomal fragments, as well as the expected ~10.4bp periodicity, resulting from steric hindrance of the helical twist of the DNA on the nucleosome surface.

**C *Control experiment for the ATAC-Seq analysis of the chromatin accessibility of the genes dysregulated by neuroinflammation in O4+ cells.*** Scatter plots representing the dispersion (logfold change) as the number of tn5 cuts per million (logCPM), for each individual analyzed peak (across 3 PBS samples and 3 IL1B samples) with permuted sample labels, created by swapping of the two closest samples: PBS-1 samples and IL1B-1 samples. In red, peaks showing differential chromatin accessibility with FDR < 0.05. Mixing PBS-1 and IL1B-1 samples led to only 1 peak, confirming that differential peaks found with correct sample labels are not statistical artefacts.

Schang et al.

**Figure S4**

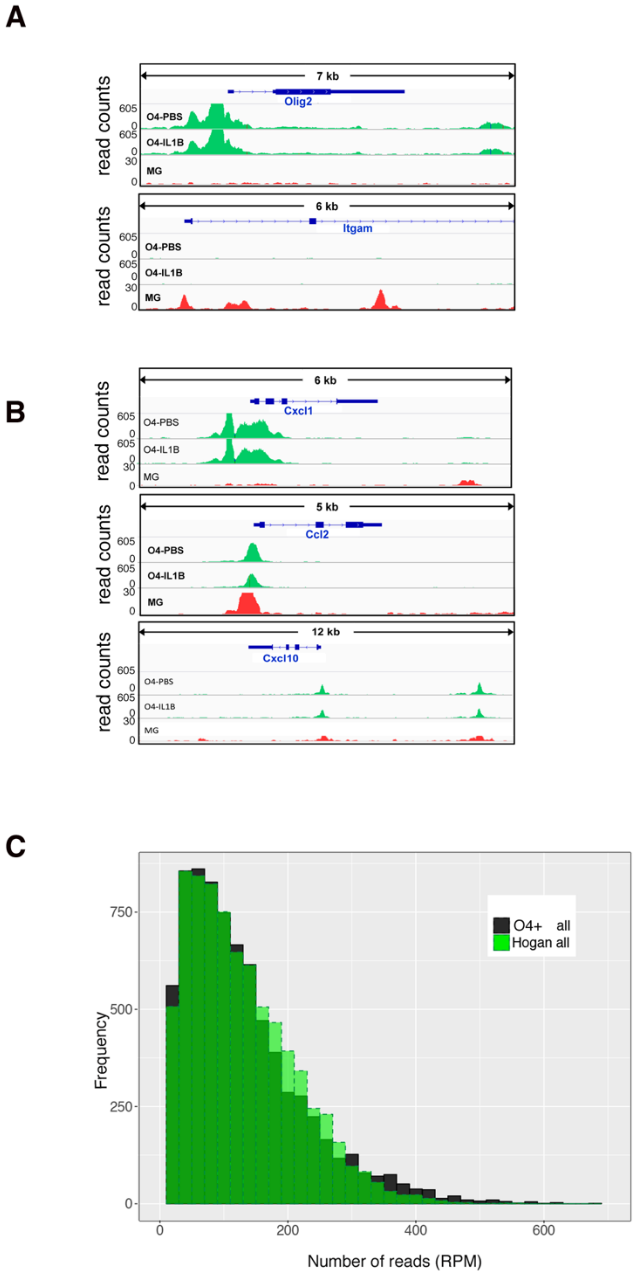


**Figure S4. Specific epigenetic priming of genes of the immune/inflammatory pathway in O4+ cells.** (related to Figure 5)

(Continued next page)

**Figure S4. Specific epigenetic priming of genes of the immune/inflammatory pathway in O4+ cells.** (related to Figure 5)

**A,B *Distinct profiles of chromatin accessibility in genes specific for OPCs and MG*.** Our O4+ cell ATAC-seq data, (green peaks; see Figure 5) were compared with ATAC-Seq data from microglia ((3) ; red peaks). Note that the peak profiles of *Cxcl1, Ccl2* or *Cxcl10* are also illustrated in Figure 5E, but, in that case, separately showing PBS1-3 and IL1B 1-3 in O4+ samples, only.

**C *Cross-species comparison confirms global similarities in the chromatin landscapes of HAEC and unstressed O4+ cell datasets*** (related to Figure 5F)
Read number distribution of peaks upon IL1B treatment in the HAEC and O4+ cell datasets. Reads were normalized for each set of peaks against the total number of reads present in the 7739 matched peaks and converted into RPM (read per million).

**Supplemental information related to Figure S4A,B. The open chromatin status of genes belonging to the innate immune/ inflammatory pathway (gene cluster C1) is not due to contamination by microglia.**

We verified that our ATAC-Seq results obtained from the O4+ isolated cell population were not due to contamination with microglia (MG). We first investigated the chromatin status of cytokine and chemokine genes in our MACS O4+ cell population, compared to microglia. i) *Itgam* is a gene that is specifically expressed in microglia and correlatively shows open chromatin conformation in this cell population; (Figure S4A, red peaks (3)). In contrast, *Itgam* exhibited a close chromatin conformation either under control PBS conditions or upon IL1B treatment in the MACS-isolated O4+ cell population (Figure S4A lower panel, absence of green peaks). This indicates that the contribution of microglia to the O4+ isolated population – if any— was negligible. Note that, conversely, *Olig2*, which is expressed by O4+ cells at P5, was found in an open chromatin conformation in MACSed O4+ cells, but not in isolated microglia (Figure S4A, green peaks in O4+ cells, but not in MG). ii) For cytokine and chemokine genes, we detected ATAC-Seq peaks with two different types profiles as illustrated in Figure S4B. i) *Cxcl1* exhibited an open chromatin state in the MACS-isolated O4+ cells population, but not in isolated MG. This implies that microglia cannot be the major contributor to the synthesis of these molecules in the MACS-isolated O4+ cell population; ii) *Ccl2* or *Cxcl10* were found in an open conformation both in MACS-isolated O4+ cells and microglia. However, as stated above, considering that our examination of peaks in other genes like *Olig2*, *Itgam*, and *Cxcl1* excluded that contamination by microglia could account for the chromatin conformation profiles of the MACS-isolated O4+ cell population, the *Ccl2* or *Cxcl10* peaks, thus most likely corresponded to the chromatin status of MACS-isolated O4+ cells, and not of MG (Figure S4B). We thus concluded that contamination of MACS-isolated O4+ cells by microglia, if any, is only very minor in this study, as it is undetectable in our ATAC-Seq experiments.

Schang et al.

**Figure S5**

**A**

**UP**

| Rank | Motif | Name | P-value | log P-pvalue | q-value (Benjamini) | # Target Sequences with Motif | % of Targets Sequences with Motif | # Background Sequences with Motif | % of Background Sequences with Motif |
|---|---|---|---|---|---|---|---|---|---|
| 1 | 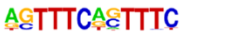 | **ISRE**(IRF)/ThioMac-LPS-Expression(GSE23622)/Homer | 1,00E-11 | -2.571e+01 | 0.0000 | 72.0 | 3.29% | 502.8 | 1.32% |
| 2 | 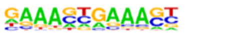 | **IRF2**(IRF)/Erythroblas-IRF2-ChIP-Seq(GSE36985)/Homer | 1,00E-09 | -2.133e+01 | 0.0000 | 109.0 | 4.98% | 1002.9 | 2.63% |
| 3 | 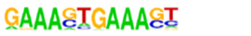 | **IRF1**(IRF)/PBMC-IRF1-ChIP-Seq(GSE43036)/Homer | 1,00E-08 | -1.892e+01 | 0.0000 | 118.0 | 5.39% | 1166.3 | 3.06% |
| 4 | 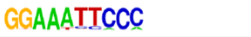 | **NFkB-p65-Rel**(RHD)/ThioMac-LPS-Expression(GSE23622)/Homer | 1,00E-04 | -1.076e+01 | 0.0021 | 57.0 | 2.61% | 546.2 | 1.43% |
| 5 | 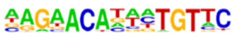 | **PGR**(NR)/EndoStromal-PGR-ChIP-Seq(GSE69539)/Homer | 1,00E-04 | -1.001e+01 | 0.0035 | 135.0 | 6.17% | 1655.1 | 4.34% |
| 6 | 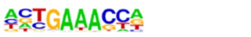 | **IRF4**(IRF)/GM12878-IRF4-ChIP-Seq(GSE32465)/Homer | 1,00E-03 | -8.091e+00 | 0.0198 | 246.0 | 11.24% | 3450.0 | 9.06% |
| 7 | 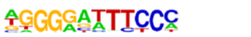 | **NFkB-p65**(RHD)/GM12787-p65-ChIP-Seq(GSE19485)/Homer | 1,00E-03 | -7.672e+00 | 0.0258 | 349.0 | 15.95% | 5127.7 | 13.46% |
| 8 | 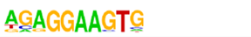 | **PU.1**(ETS)/ThioMac-PU.1-ChIP-Seq(GSE21512)/Homer | 1,00E-03 | -7.299e+00 | 0.0327 | 346.0 | 15.81% | 5107.0 | 13.41% |
| 9 | 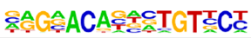 | **GRE**(NR),IR3/A549-GR-ChIP-Seq(GSE32465)/Homer | 1,00E-03 | -7.297e+00 | 0.0327 | 102.0 | 4.66% | 1274.8 | 3.35% |
| 10 | 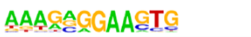 | **SpiB**(ETS)/OCILY3-SPIB-ChIP-Seq(GSE56857)/Homer | 1,00E-03 | -7.060e+00 | 0.0332 | 174.0 | 7.95% | 2381.8 | 6.25% |
| 11 | 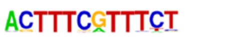 | **T1ISRE**(IRF)/ThioMac-Ifnb-Expression/Homer | 1,00E-02 | -5.778e+00 | 0.1089 | 12.0 | 0.55% | 81.0 | 0.21% |
| 12 | 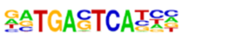 | **Jun-AP1**(bZIP)/K562-cJun-ChIP-Seq(GSE31477)/Homer | 1,00E-02 | -5.586e+00 | 0.1209 | 129.0 | 5.90% | 1764.0 | 4.63% |
| 13 | 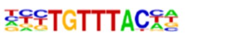 | **FOXP1**(Forkhead)/H9-FOXP1-ChIP-Seq(GSE31006)/Homer | 1,00E-02 | -5.484e+00 | 0.1236 | 199.0 | 9.10% | 2874.6 | 7.55% |
| 14 | 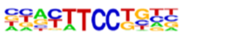 | **Etv2**(ETS)/ES-ER71-ChIP-Seq(GSE59402)/Homer(0.967) | 1,00E-02 | -5.312e+00 | 0.1363 | 616.0 | 28.15% | 9790.6 | 25.70% |
| 15 | 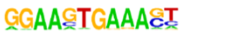 | **PU.1:IRF8**(ETS:IRF)/pDC-Irf8-ChIP-Seq(GSE66899)/Homer | 1,00E-02 | -5.274e+00 | 0.1363 | 128.0 | 5.85% | 1766.9 | 4.64% |
| 16 | 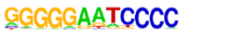 | **NFkB-p50,p52**(RHD)/Monocyte-p50-ChIP-Chip(Schreiber_et_al.)/Homer | 1,00E-02 | -5.184e+00 | 0.1363 | 126.0 | 5.76% | 1740.2 | 4.57% |
| 17 | 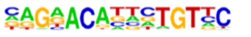 | **GRE**(NR),IR3/RAW264.7-GRE-ChIP-Seq(Unpublished)/Homer | 1,00E-02 | -4.809e+00 | 0.1857 | 147.0 | 6.72% | 2093.4 | 5.50% |
| 18 | 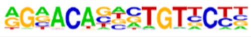 | **ARE**(NR)/LNCAP-AR-ChIP-Seq(GSE27824)/Homer | 1,00E-02 | -4.777e+00 | 0.1857 | 170.0 | 7.77% | 2461.1 | 6.46% |

**B**

**ALL**

| Rank | Motif | Name | P-value | log P-pvalue | q-value (Benjamini) | # Target Sequences with Motif | % of Targets Sequences with Motif | # Background Sequences with Motif | % of Background Sequences with Motif |
|---|---|---|---|---|---|---|---|---|---|
| 1 | 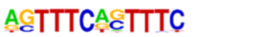 | **ISRE**(IRF)/ThioMac-LPS-Expression(GSE23622)/Homer | 1,00E-08 | -1.915e+01 | 0.0000 | 83.0 | 2.67% | 492.7 | 1.33% |
| 2 | 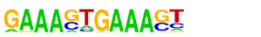 | **IRF1**(IRF)/PBMC-IRF1-ChIP-Seq(GSE43036)/Homer | 1,00E-06 | -1.452e+01 | 0.0001 | 147.0 | 4.73% | 1148.0 | 3.09% |
| 3 | 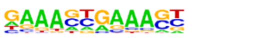 | **IRF2**(IRF)/Erythroblas-IRF2-ChIP-Seq(GSE36985)/Homer | 1,00E-05 | -1.343e+01 | 0.0002 | 129.0 | 4.15% | 996.4 | 2.68% |
| 4 | 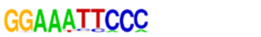 | **NFkB-p65-Rel**(RHD)/ThioMac-LPS-Expression(GSE23622)/Homer | 1,00E-04 | -9.613e+00 | 0.0065 | 73.0 | 2.35% | 538.6 | 1.45% |
| 5 | 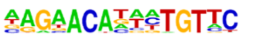 | **PGR**(NR)/EndoStromal-PGR-ChIP-Seq(GSE69539)/Homer | 1,00E-03 | -7.792e+00 | 0.0320 | 174.0 | 5.60% | 1604.2 | 4.31% |
| 6 | 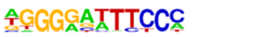 | **NFkB-p65**(RHD)/GM12787-p65-ChIP-Seq(GSE19485)/Homer | 1,00E-02 | -5.940e+00 | 0.1698 | 481.0 | 15.47% | 5097.2 | 13.71% |
| 7 | 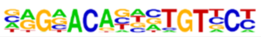 | **GRE**(NR),IR3/A549-GR-ChIP-Seq(GSE32465)/Homer | 1,00E-02 | -5.585e+00 | 0.2075 | 133.0 | 4.28% | 1252.3 | 3.37% |
| 8 | 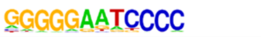 | **NFkB-p50,p52**(RHD)/Monocyte-p50-ChIP-Chip(Schreiber_et_al.)/Homer | 1,00E-02 | -5.013e+00 | 0.3219 | 178.0 | 5.73% | 1763.5 | 4.74% |
| 9 | 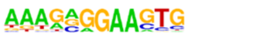 | **SpiB**(ETS)/OCILY3-SPIB-ChIP-Seq(GSE56857)/Homer | 1,00E-02 | -4.708e+00 | 0.3880 | 230.0 | 7.40% | 2353.2 | 6.33% |

**Figure S5. TFBS motifs identified in significant ATAC-seq peaks in the vicinity of TSS (+/-8 kb)**, adjacent to upregulated genes (UP; **A**) and all differentially regulated genes (ALL; **B**), using HOMER known motifs. (related to Figure 6)

## LIST AND LEGENDS OF TABLES AND OF SUPPLEMENTAL MATERIAL

**Table S1. List of differentially expressed genes per relevant gene cluster.**
List of the differentially expressed genes from the microarray analysis of Agilent microarray probes for each cluster. The corresponding Refseq gene ID is indicated.

**Table S2. GO-term Biological Pathway analysis of the genes belonging to Cluster C1.**
GO-term Biological Pathway enrichment analysis was performed using David6.8 on C1 cluster from the in the top 5 most statistically significant pathways from the microarray analysis (Table S1).

**Table S3. Single-cell analysis performed from data obtained from mouse cortices at P7**, published by Jin et al. (4).

**Table S4. Alignment statistics of ATAC-seq.**
The alignment statistics of the samples is in line with what is expected from ATAC-Seq samples. Losing in the region of 10% of reads to mitochondrial alignment is normal for this type of data.

**Table S5. Coordinates of the 213,246 peaks (mm10) detected in PBS and/or IL1B samples**
MACS2 peak calling was run separately on PBS and IL1B pooled samples (n=3/group). The two resulting peak files (almost 200,000 peaks in each condition) were merged and the mm10 blacklist removed, leading to a list of 213,246 peaks detected in at least one condition (mm10 coordinates).

**Table S6. List and annotation of the 524 differentially accessible peaks**
Reads were counted in each of the 213,246 peaks (Table S2) for each sample individually (3 PBS and 3 IL1B samples). Comparison and statistical analysis with EdgeR (exact test and FDR by Benjamini-Hochberg method) identified 524 peaks with differential accessibility (FDR<0.05). Peaks were annotated using HOMER annotatePeaks.

**Table S7. Among the 27 regions, located within +/- 8kb around TSS, that are associated differential opening and closing of chromatin conformation** (highlighted in yellow in the list)**, 19 were involved in the immune system and inflammatory response pathways** (in red). We found 1250 UP genes associated to 2466 peaks.

**Table S8. Hogan (HAECS) (list of human gene names and the corresponding orthologue genes in mice, that have been used for the cross-species comparison.**

**Table S9. ATAC-Seq peaks associated with upregulated genes and revealing the existence of paired TFBS motifs**

**Table S10. GO-term Biological Pathway analysis of the genes belonging to Cluster C2.**

**Table S11. GO-term Biological Pathway analysis of the genes belonging to Cluster C3.**

**Table S12. GO-term Biological Pathway analysis of the genes belonging to Cluster C4.**

**Table S13: List of the RT-qPCR primers**

**Jupyter notebook: ATAC-seq workflow**
Detailed and explained bioinformatics workflow used for the analysis of ATAC-seq dataset.

**RMarkDown workflow for GSE157977_scRNA_ctrl**
Detailed and explained bioinformatics

______________

## Supplementary references

1. Krishnan ML, Van Steenwinckel J, Schang AL, Yan J, Arnadottir J, Le Charpentier T, et al. Integrative genomics of microglia implicates DLG4 (PSD95) in the white matter development of preterm infants. Nat Commun. déc 2017;8(1):428.

2. Van Steenwinckel J, Schang AL, Krishnan ML, Degos V, Delahaye-Duriez A, Bokobza C, et al. Decreased microglial Wnt/β-catenin signalling drives microglial pro-inflammatory activation in the developing brain. Brain. 1 déc 2019;142(12):3806-33.

3. Matcovitch-Natan O, Winter DR, Giladi A, Vargas Aguilar S, Spinrad A, Sarrazin S, et al. Microglia development follows a stepwise program to regulate brain homeostasis. Science. 19 août 2016;353(6301):aad8670.

4. Jin X, Simmons SK, Guo A, Shetty AS, Ko M, Nguyen L, et al. In vivo Perturb-Seq reveals neuronal and glial abnormalities associated with autism risk genes. Science. 27 nov 2020;370(6520):eaaz6063.
