# Supplementary material for: Epigenetic priming of immune/inflammatory pathways activation and abnormal activity of cell cycle pathway in a perinatal model of white matter injury
Source: Cell Death Dis. 2022 Dec 13;13(12):1038. doi: 10.1038/s41419-022-05483-4 (PMC9748018; doi:10.1038/s41419-022-05483-4)
Supplement: Supplementary file 5 — Table S4 [file 41419_2022_5483_MOESM5_ESM.docx]

## **Table S4. Alignment Statistics of ATAC-Seq data**

| Paired |  | **PBS1** | | **PBS2** | | **PBS3** | | **IL1B1** | | **IL1B2** | | **IL1B3** | |
| --- | --- | --- | --- | --- | --- | --- | --- | --- | --- | --- | --- | --- | --- |
| **Total Reads** |  | 73 686 516 | | 67 049 336 | | 79 305 947 | | 74 117 441 | | 61 622 256 | | 76 360 806 | |
|  |  |  | |  | |  | |  | |  | |  | |
| **Removing Mitochondria** | **Remaining Reads** | 66 505 362 | | 60 228 153 | | 71 141 195 | | 66 632 160 | | 55 217 126 | | 66 962 284 | |
|  | **% of Total Removed** | 9.75% | | 10.17% | | 10.30% | | 10.10% | | 10.39% | | 12.31% | |
|  |  |  | |  | |  | |  | |  | |  | |
| **% Reads Remaining to Align to Nuclear** |  | 90.25% | | 89.83% | | 89.70% | | 89.90% | | 89.61% | | 87.69% | |
| **Nuclear Only** | **Con 1 time only** | 57 751 458 | | 52 192 349 | | 62 471 914 | | 56 958 297 | | 47 542 920 | | 58 362 195 | |
|  | **Con >1 time** | 340 054 | | 325 951 | | 371 952 | | 348 211 | | 320 270 | | 351 148 | |
|  | **Discon 1 time** | 47 160 | | 34 979 | | 42 034 | | 43 352 | | 31 631 | | 39 905 | |
|  | **Without mates 1** | 541 607 | | 526 165 | | 592 143 | | 522 936 | | 491 098 | | 569 201 | |
|  | **Without mates >1** | 340 054 | | 325 951 | | 371 952 | | 348 211 | | 320 270 | | 351 148 | |
|  | ***Total All Alignments*** | *59 020 333* | | *53 405 395* | | *63 849 995* | | *58 221 007* | | *48 706 189* | | *59 673 597* | |
|  |  |  | |  | |  | |  | |  | |  | |
|  | **% of total reads** | 80.10% | | 79.65% | | 80.51% | | 78.55% | | 79.04% | | 78.15% | |
|  |  |  | |  | |  | |  | |  | |  | |
|  |  |  | |  | |  | |  | |  | |  | |
| **Nuclear Reads Mapping 1 time only** | | 58 340 225 | 52 753 493 | | 63 106 091 | | 57 524 585 | | 48 065 649 | | 58 971 301 | |  |
|  |  |  | |  | |  | |  | |  | |  | |
| **% of Total Reads Mapping to nucleus 1 time only** | | 79.17% | 78.68% | | 79.57% | | 77.61% | | 78.00% | | 77.23% | |  |

The alignment statistics of the samples is in line with what are expected from ATAC-seq samples. Losing in the region of 10% of reads to mitochondrial alignment is usual for this type of data.
