# Supplementary material for: Epigenetic priming of immune/inflammatory pathways activation and abnormal activity of cell cycle pathway in a perinatal model of white matter injury
Source: Cell Death Dis. 2022 Dec 13;13(12):1038. doi: 10.1038/s41419-022-05483-4 (PMC9748018; doi:10.1038/s41419-022-05483-4)
Supplement: Supplementary file 14 — Table S13 [file 41419_2022_5483_MOESM14_ESM.pdf]

	Forward	Reverse
Ccl2	CATCCACGTGTTGGCTCA	TCATTGGGATCATCTTGCTG
Ccl3	TTTTGAAACCAGCAGCCTTT	CTGCCTCCAAGACTCTCAGG
Ccl4	CCCACTTCCTGCTGTTTCTC	GTCTGCCTCTTTTGGTCAGG
Ccl5	GTGCCCACGTCAAGGAGTAT	CCCACTTCTTCTCTGGGTTG
Ccr1	AGGCCCAGAAACAAAGTCTG	TTGTGGGGTAGGCTTCTGTG
Ccr2	GCCAGGACAGTTACCTTTGG	TTCCTGGTAGAGAGGCAAACA
Cnp	AGACAGCGTGGCGACTAGACT	GGGCTTCAGCTTCTTCAGGT
Cxcl1	GCACCCAAACCGAAGTCATA	AGGTGCCATCAGAGCAGTCT
Cxcl10	GCTGCAACTGCATCCATATC	GGATTCAGACATCTCTGCTCAT
Cxcl12	AGAGCCAACGTCAAGCATCT	TAATTTCGGGTCAATGCACA
Cxcl13	GGAGAGCGACACAGCAGAAC	ACCACCTCTCCCATGTCATC
Cxcl16	GGAGAGCGACACAGCAGAAC	ACCACCTCTCCCATGTCATC
Cxcl2	CAAGGGCGGTCAAAAAGTT	TCCAGGTCAGTTAGCCTTGC
Cxcl9	ACGGAGATCAAACCTGCCTA	TTTCCCCCTCTTTTGCTTTT
Gapdh	GGCCTTCCGTGTTCCTAC	TGTCATCATACTTGGCAGGTT
Gfap	AAGCCAAGCACGAAGCTAAC	CTCCTGGTAACTGGCCGACT
Id2	CTGGACTCGCATCCCACTAT	CGACATAAGCTCAGAAGGGAAT
Il1a	GACGGCTGAGTTTCAGTGAG	TAAGGTGCTGATCTGGGTTG
Il1b	GGGCCTCAAAGGAAAGATTC	TCTTCTTTGGGTATTGCTTGG
Il1r1	CAAGCTGTTCATTTATGGAAGG	ATCAGCCTCCTGCTTTTCTTT
Il1r2	TAAATGTGTTGCCTCGAATCC	CTCCAGGAGAACGTGGAAGA
Il6	CAAAGCCAGAGTCCTTCAGA	GCCACTCCTTCTGTGACTCC
Il6ra	CGTTTGGGTTGCTTCTCTGT	GTGGAGGAGAGGTCGTCTTG
Itgam	CTGGTGCTCTTGGCTCTCAT	GGCAGCTTCATTCATCATGT
Mbp	CCGGACCCAAGATGAAAAC	CTTGGGATGGAGGTGGTGT
Mog	AAGAGGCAGCAATGGAGTTG	GACCTGCAGGAGGATCGTAG
Pdgfra	GACGTTCAAGACCAGCGAGTT	CAGTCTGGCGTGCGTCC
Rbfox3(NeuN)	CAGATATGCTCAGCCAGCAG	CGATGCTGTAGGTTGCTGTG
Rpl13a	ACAGCCACTCTGGAGGAGAA	GAGTCCGTTGGTCTTGAGGA
