# Supplementary material for: Epigenetic priming of immune/inflammatory pathways activation and abnormal activity of cell cycle pathway in a perinatal model of white matter injury
Source: Cell Death Dis. 2022 Dec 13;13(12):1038. doi: 10.1038/s41419-022-05483-4 (PMC9748018; doi:10.1038/s41419-022-05483-4)
Supplement: Supplementary file 15 — ATAC-seq workflow [file 41419_2022_5483_MOESM15_ESM.pdf]

ATACseq workflow

Trimming

The fastq file were trimmed using Trimmomatic v0.33 to remove any adapter sequences in the reads caused by read through associated with DNA fragments shorter in size than the read length being sequenced. Trimmomatic software were downloaded and installed from http://www.usadellab.org/cms/index.php?page=trimmomatic (http://www.usadellab.org/cms/index.php?page=trimmomatic)

In [ ]:

```
java-jar path/Trimmomatic-0.33/trimmomatic-0.33.jar PE-threads 12 reads_path/A_1.fastq.
gz reads_path/A_2.fastq.gz trimmed_files/A_1_tr.fastq trimmed_files/A_1_tr_unpaired.fas
tq trimmed_files/A_2_tr.fastq   trimmed_files/A_2_tr_unpaired.fastq ILLUMINACLIP:Nexter
aPE-PE.fa:2:30:10:1:true TRAILING:3 SLIDINGWINDOW:4:15
```

Quality control and compression of the unpaired reads

In [ ]:

```
FastQC/fastqc –o output_directory path_to_data/input_file_name
```

In [ ]:

```
gzip A_1_tr_unpaired.fastq A_2_tr_unpaired.fastq
```

Alignment

In order to align reads to each cellular compartment it is necessary to build an index for each compartment, and then only the reads from the compartment of interest are saved.

Build command within Bowtie 2 is ran to create an index for the mitochondria and for the nucleus

In [ ]:

```
path_to_bowtie2/bowtie2-build –f chrM.fa species_vn_chrM
path_to_bowtie2/bowtie2-build –f chr1.fa, chr2.fa, …, chrLast.fa mouse_vn_chrN
```

Mitochondrial DNA is removed

In [ ]:

```
path_to_bowtie2/bowtie2 -p 12 -X 2000 --very-sensitive -x mouse_vn_chrM -1 trimmed_file
s /A_1_tr.fastq.gz -2 trimmed_files /A_2_tr.fastq.gz -S temp_files/A_tempM.sam --un-con
c temp_files/A_noM_fastq
```

The temporary file containing the aligned Mitochondrial read is deleted and the resulting Fastq file is compressed

In [ ]:

```
rm temp_files/A_tempM.sam
gzip temp_files/A_noM_fastq.1 temp_files/A_noM_fastq.2
```

The cleaned fastq files is used as the input to map the remaining reads to the nuclear mouse genome

The Bowtie2 log for the alignment is saved to track the number of reads that aligns to each cellular compartment and then the temporary Fastq file is deleted, the sam file generated is converted into a bam file and the SAM file is deleted if it is no longer required.

In [ ]:

```
path_to_bowtie2/bowtie2 -p 12 -X 2000 --very-sensitive -x mouses_vn_chrN -1 temp_files/
A_Nuclear_fastq.1.gz -2 temp_files/A_Nuclear_fastq.2.gz -S aligned/ A_Nuclear sam
rm temp_files/A_Nuclear_fastq.*.gz
samtools-1.2/samtools view -b -S path_to_file/A_alignedX.sam -o 10 path_to_file/A_align
edX_unsorted.bam
rm path_to_file/A_alignedX.sam
```

The aligned reads BAM file is sorted and indexed, the **unsorted** BAM file is deleted if it is no longer required.

In [ ]:

```
samtools-1.2/samtools sort path_to_file/A_alignedX_unsorted.bam path_to_file/ A_aligned
X.bam
rm path_to_file/A_alignedX_unsorted.bam
samtools-1.2/samtools index path_to_file/A_alignedX.bam
```

Calculation of BAM flag distribution by using Samtools (*Use Table 2 of Kate's paper as a template to translate the BAM flag codes into a format that is easier to interpret*) and visualise

In [ ]:

```
path_to_samtools/samtools-1.2/samtools view path_to_file/A_alignedX.bam | awk '{print
 $2}' | sort -n | uniq -c | sed 's/^ *//g' > path_to_output/A_alignedX_bamflags.txt
```

Quality control

Creation of the MAPQ Score distribution by using Samtools

In [ ]:

```
path_to_samtools/samtools-1.2/samtools view -f2 path_to_file/aligned_reads.bam | awk
'{print  $5}' | sort -n | uniq -c | sed 's/^ *//g' > path_to_output/A_alignedX_mapq.txt
```

Calculation of the insert size distribution for high quality, properly mapped reads for visualisaion

In [ ]:

```
samtools-1.2/samtools view -f2 –q22 path_to_file/A_alignedX_.bam | cut -f 9|sed 's/^
-//' | sort -n | uniq -c > path_to_output/A_alignedX__is_summary.txt
```

Creation of a BAM file that contains the high quality, properly mapped reads only

In [ ]:

```
samtools-1.2/samtools view -f2 –q22 path_to_file/aligned_reads.bam > path_to_output/A_a
lignedX_filter.bam
```

New FastQC in order to verify the improvement

In [ ]:

```
FastQC/fastqc –o output_directory path_to_data/input_file_name
```

Merging files

Files were merged

Control sample files (1A,2B,4B) are merged and named : mergeCtrl_Trim.bam

IL1 treated sample files (7A,9A,8B) are merged and named : mergeIL1_Trim.bam

In [ ]:

```
samtools merge mergeCtrl_Trim.bam eg1ATrim_sorted_deduplicated.bam eg2BTrim_sorted_dedu
plicated.bam eg4BTrim_sorted_deduplicated.bam
samtools merge mergeIL1_Trim.bam eg7ATrim_sorted_deduplicated.bam eg8BTrim_sorted_dedup
licated.bam eg9ATrim_sorted_deduplicated.bam
```

Peak calling with MACS2 for the 2 series

In [ ]:

```
macs2 callpeak -t mergeCtrl_Trim.bam -f BAM -g 1.87e9 -q 0.05 -n mergeCtrl_Trim.bed
macs2 callpeak -t mergeIL1_Trim.bam -f BAM -g 1.87e9 -q 0.05 -n mergeIL1_Trim.bed
```

the 2 bedfiles are combined in a text file (named Ctrl_IL1_Trim_peaks.txt) the output file is named Ctrl_IL1_Trim_peaks.bed

In [ ]:

```
bedtools merge -i Ctrl_IL1_Trim_peaks.txt > Ctrl_IL1_Trim_peaks.bed
```

blacklisted peaks are removed using bedtools subtractbed

BLACKLIST="http://mitra.stanford.edu/kundaje/akundaje/release/blacklists/mm10-mouse/mm10.blacklist.bed.gz (http://mitra.stanford.edu/kundaje/akundaje/release/blacklists/mm10-mouse/mm10.blacklist.bed.gz)" the output file is named Ctrl_IL1_Trim_BL.bed

In [ ]:

```
subtractBed -A -a Ctrl_IL1_Trim_peaks.bed -b blacklist
```

in order to determine the number of reads in each sample per peak we used Bedtools coverage

In [ ]:

```
bedtools coverage -a Ctrl_IL1_Trim_BL.bed -b eg1ATrim_sorted_deduplicated.bam -counts > cov_1A.bed
bedtools coverage -a Ctrl_IL1_Trim_BL.bed -b eg2BTrim_sorted_deduplicated.bam -counts > cov_2B.bed
bedtools coverage -a Ctrl_IL1_Trim_BL.bed -b eg4BTrim_sorted_deduplicated.bam -counts > cov_4B.bed
bedtools coverage -a Ctrl_IL1_Trim_BL.bed -b eg7ATrim_sorted_deduplicated.bam -counts > cov_7A.bed
bedtools coverage -a Ctrl_IL1_Trim_BL.bed -b eg8BTrim_sorted_deduplicated.bam -counts > cov_8B.bed
bedtools coverage -a Ctrl_IL1_Trim_BL.bed -b eg9ATrim_sorted_deduplicated.bam -counts > cov_9A.bed
```

number of reads are normalized to the library size and combined in a text file named Count-peaks-all-norm.txt

the analysis is done using edgeR package

In [ ]:

```
library(edgeR)
x<-read.delim("E:/Count-peaks-all-norm.txt")
group <- factor(c(1,1,1,2,2,2))
y <- DGEList(counts=x[,2:7],group=group)
y <- calcNormFactors(y)
y$samples
y <- estimateCommonDisp(y)
sqrt(y$common.disp)
plotBCV(y)
plotMDS(y, method="bcv")
y <- estimateTagwiseDisp(y)
et <- exactTest(y)
topTags(et)
Z=summary(de <- decideTestsDGE(et, p=0.05, adjust="BH"))
detags <- rownames(y)[as.logical(de)]
plotSmear(et, ylim=c(-6,6), de.tags=detags)
abline(h = c(-2, 2), col = "blue")
et.df = as.data.frame(do.call(rbind, et))
write.table(et.df,"results",quote=FALSE,row.names=TRUE,sep="\t")
```
