# Supplementary material for: Epigenetic priming of immune/inflammatory pathways activation and abnormal activity of cell cycle pathway in a perinatal model of white matter injury
Source: Cell Death Dis. 2022 Dec 13;13(12):1038. doi: 10.1038/s41419-022-05483-4 (PMC9748018; doi:10.1038/s41419-022-05483-4)
Supplement: Supplementary file 16 — RMarkDown workflow for scAnalysis fig3&S3 [file 41419_2022_5483_MOESM16_ESM.html]

GSE157977\_scRNA\_ctrl


# GSE157977\_scRNA\_ctrl

```
knitr::opts_chunk$set(
  tidy = TRUE,
  tidy.opts = list(width.cutoff = 95),
  message = FALSE,
  warning = FALSE,
  time_it = TRUE
)
```

```
# install.packages('hdf5r')

# load R packages
library(dplyr)
library(Seurat)
library(ggplot2)
```

#### Read data

```
# Load the datasets
control <- Read10X_h5(paste0(data_path, "GSM4782559_UMI.counts.GFP.h5"), use.names = T)
```

# Seurat

### Create Seurat object

```
control.data <- CreateSeuratObject(control, project = "control", min.cells = 3, min.features = 200)

rm(control)
```

#### Calculate mitochondrial proportion

```
head(control.data@meta.data)
```

```
##                    orig.ident nCount_RNA nFeature_RNA
## AAACCTGAGGCTACGA-1    control      33102         6228
## AAACCTGAGGCTATCT-1    control       6492         2257
## AAACCTGAGGGTATCG-1    control       2832          781
## AAACCTGAGTGTCCCG-1    control      27921         5649
## AAACCTGCAGAGCCAA-1    control       2980         1367
## AAACCTGCATGTAAGA-1    control       1539          465
```

```
control.data[["percent.mt"]] <- PercentageFeatureSet(control.data, pattern = "^mt-")
```

```
# Visualize QC metrics as a violin plot
VlnPlot(control.data, features = c("nFeature_RNA", "nCount_RNA", "percent.mt"), ncol = 3)
```

```
QC.plot1 <- FeatureScatter(control.data, feature1 = "nCount_RNA", feature2 = "percent.mt")
QC.plot2 <- FeatureScatter(control.data, feature1 = "nCount_RNA", feature2 = "nFeature_RNA")
QC.plot1 + QC.plot2
```

```
control.data <- subset(control.data, subset = nFeature_RNA > 200 & nFeature_RNA < 9000 & percent.mt <
    15)
```

# Normalizing the data

```
# Normalise the data by total features per cell

control.data <- NormalizeData(control.data, normalization.method = "LogNormalize", scale.factor = 10000)
```

# Identification of highly variable features (feature selection)

```
control.data <- FindVariableFeatures(control.data, selection.method = "vst", nfeatures = 2000)

# Identify the 10 most highly variable genes
top10 <- head(VariableFeatures(control.data), 10)

# Plot variable features with and without labels
VF.plot1 <- VariableFeaturePlot(control.data)
VF.plot2 <- LabelPoints(plot = VF.plot1, points = top10, repel = TRUE)
VF.plot1 + VF.plot2
```

---

# Scaling the data

```
# scale data and remove unwanted sources of variation

control.data <- ScaleData(control.data, vars.to.regress = "percent.mt")
```

---

# Perform linear dimensional reduction

```
control.data <- RunPCA(control.data, features = VariableFeatures(object = control.data))
```

```
# Examine and visualize PCA results a few different ways
print(control.data[["pca"]], dims = 1:5, nfeatures = 5)
```

```
## PC_ 1 
## Positive:  Syp, Grin2b, Snhg11, L1cam, Syt1 
## Negative:  Sepp1, Sparc, Cyba, Lmo2, Eva1b 
## PC_ 2 
## Positive:  Stmn2, Tubb3, Mef2c, Stmn1, Stmn3 
## Negative:  Odf3b, Rsph1, 2410004P03Rik, Foxj1, Dynlrb2 
## PC_ 3 
## Positive:  C1qb, C1qa, C1qc, Fcer1g, Tyrobp 
## Negative:  Col4a2, Col4a1, Cldn5, Foxq1, Flt1 
## PC_ 4 
## Positive:  Laptm5, C1qb, C1qa, Cx3cr1, Rnase4 
## Negative:  Bcan, Fabp7, Aldoc, Ednrb, Tst 
## PC_ 5 
## Positive:  Tubb3, Stmn2, Stmn3, Stmn1, Hbb-bs 
## Negative:  Atp1a2, Tsc22d4, Sdc4, Socs3, Ctsb
```

```
VizDimLoadings(control.data, dims = 1:2, reduction = "pca")
```

```
DimPlot(control.data, reduction = "pca")
```

```
DimHeatmap(control.data, dims = 1:15, cells = 500, balanced = TRUE)
```

# Determine the ‘dimensionality’ of the dataset

```
control.data <- JackStraw(control.data, num.replicate = 100)
control.data <- ScoreJackStraw(control.data, dims = 1:20)
```

```
JackStrawPlot(control.data, dims = 1:15)
```

```
ElbowPlot(control.data)
```

---

# Cluster the cells

```
control.data <- FindNeighbors(control.data, dims = 1:8)
control.data <- FindClusters(control.data, resolution = 0.5)
```

```
## Modularity Optimizer version 1.3.0 by Ludo Waltman and Nees Jan van Eck
## 
## Number of nodes: 3080
## Number of edges: 86547
## 
## Running Louvain algorithm...
## Maximum modularity in 10 random starts: 0.9211
## Number of communities: 20
## Elapsed time: 0 seconds
```

```
# Look at cluster IDs of the first 5 cells
head(Idents(control.data), 5)
```

```
## AAACCTGAGGCTACGA-1 AAACCTGAGGCTATCT-1 AAACCTGAGTGTCCCG-1 AAACCTGCAGAGCCAA-1 
##                  5                  7                  8                  8 
## AAACCTGGTCTCTCTG-1 
##                  3 
## Levels: 0 1 2 3 4 5 6 7 8 9 10 11 12 13 14 15 16 17 18 19
```

---

# Run non-linear dimensional reduction (tSNE)

```
control.data <- RunTSNE(control.data, dims = 1:8, seed.use = 1986)
```

```
DimPlot(control.data, reduction = "tsne", label = TRUE)
```

---

# Finding differentially expressed features (cluster biomarkers)

```
# find markers for every cluster compared to all remaining cells, report only the positive
# ones
control.data.all.markers <- FindAllMarkers(control.data, only.pos = TRUE, min.pct = 0.25, logfc.threshold = 0.25)
control.data.all.markers %>%
    group_by(cluster) %>%
    top_n(n = 20, wt = avg_log2FC)
```

```
## # A tibble: 400 × 7
## # Groups:   cluster [20]
##       p_val avg_log2FC pct.1 pct.2 p_val_adj cluster gene   
##       <dbl>      <dbl> <dbl> <dbl>     <dbl> <fct>   <chr>  
##  1 5.32e-99      0.923 0.98  0.972  9.82e-95 0       mt-Atp6
##  2 7.80e-74      0.556 0.096 0.606  1.44e-69 0       Ap3s1  
##  3 4.60e-64      0.820 0.95  0.966  8.49e-60 0       mt-Co3 
##  4 5.63e-51      0.622 0.185 0.687  1.04e-46 0       mt-Nd5 
##  5 2.69e-47      0.930 0.197 0.691  4.96e-43 0       Comt   
##  6 2.24e-46      0.626 0.083 0.457  4.14e-42 0       Apc    
##  7 6.04e-46      0.787 0.911 0.929  1.12e-41 0       mt-Nd1 
##  8 2.34e-38      0.728 0.853 0.907  4.32e-34 0       mt-Co2 
##  9 2.57e-36      0.599 0.066 0.367  4.75e-32 0       Palm   
## 10 4.37e-36      0.575 0.146 0.516  8.07e-32 0       Mgst3  
## # … with 390 more rows
```

```
# find all markers distinguishing cluster 10 from clusters 1, 9 and 12
cluster10.markers <- FindMarkers(control.data, ident.1 = 10, ident.2 = c(1, 9, 12), only.pos = TRUE,
    min.pct = 0.25, logfc.threshold = 0.25)
head(cluster10.markers, n = 30)
```

```
##                      p_val avg_log2FC pct.1 pct.2    p_val_adj
## Pdgfra        1.060861e-90  3.1590119 0.693 0.002 1.959410e-86
## Lhfpl3        5.778974e-80  2.8137148 0.727 0.024 1.067377e-75
## Cspg4         6.554804e-65  1.4196216 0.568 0.013 1.210672e-60
## Nxph1         1.900053e-61  1.5944883 0.534 0.009 3.509398e-57
## 3110035E14Rik 2.622914e-60  1.8306052 0.727 0.057 4.844522e-56
## Pcdh15        8.795074e-60  1.6822794 0.523 0.011 1.624450e-55
## Sapcd2        1.801874e-55  1.5677019 0.705 0.072 3.328062e-51
## Gpr17         2.069766e-53  2.4848847 0.693 0.064 3.822857e-49
## Sox10         5.065992e-52  1.6511948 0.648 0.053 9.356887e-48
## Lrrtm3        1.044854e-50  1.0460688 0.545 0.031 1.929845e-46
## Plppr1        1.551779e-50  1.4985399 0.557 0.035 2.866136e-46
## Cacng4        1.649580e-48  2.0325286 0.864 0.212 3.046774e-44
## Ptpre         3.836620e-45  1.3185253 0.568 0.042 7.086237e-41
## Csmd3         7.477806e-43  1.2030309 0.420 0.017 1.381151e-38
## C1ql3         1.317052e-41  1.1327944 0.443 0.024 2.432596e-37
## 1700086L19Rik 1.491805e-41  1.4397009 0.523 0.048 2.755364e-37
## Resp18        8.745877e-41  1.0808566 0.398 0.015 1.615364e-36
## Anks1b        2.112566e-40  1.4977166 0.636 0.087 3.901910e-36
## Rprm          2.490926e-40  1.5195068 0.648 0.103 4.600740e-36
## Ube2c         1.467016e-39  2.5820475 0.398 0.017 2.709578e-35
## Lmnb1         5.132339e-39  1.5194371 0.591 0.083 9.479431e-35
## Ly6h          8.782938e-39  1.5973768 0.761 0.175 1.622209e-34
## Qpct          2.219406e-38  1.0121161 0.420 0.024 4.099242e-34
## C1ql1         4.183895e-38  2.8809049 0.830 0.269 7.727653e-34
## Fam64a        6.167103e-38  1.1260982 0.318 0.004 1.139064e-33
## Cdo1          3.732872e-37  2.4153119 0.761 0.204 6.894614e-33
## Aurkb         9.927921e-37  0.8275427 0.284 0.000 1.833687e-32
## Olig2         3.326620e-36  2.3858569 0.875 0.344 6.144267e-32
## Tacc2         1.043539e-35  1.2100610 0.534 0.070 1.927416e-31
## Ccna2         1.442236e-35  1.2706156 0.341 0.011 2.663809e-31
```

```
VlnPlot(control.data, features = c("Mbp", "Pdgfra", "Olig2", "Sox10", "Gpr17"))
```

```
VlnPlot(control.data, features = c("Mbp", "Pdgfra", "Olig2", "Sox10", "Gpr17"), slot = "counts",
    log = TRUE)
```

```
FeaturePlot(control.data, features = c("Mbp", "Pdgfra", "Olig2", "Sox10", "Gpr17"))
```

```
VlnPlot(control.data, features = c("Ccl2", "Cxcl1", "Cxcl10"))
```

```
FeaturePlot(control.data, features = "Ccl2") + theme(axis.text.x = element_blank(), axis.text.y = element_blank(),
    axis.ticks = element_blank()) + xlab("tSNE 1") + ylab("tSNE 2")
```

```
FeaturePlot(control.data, features = "Cxcl1") + theme(axis.text.x = element_blank(), axis.text.y = element_blank(),
    axis.ticks = element_blank()) + xlab("tSNE 1") + ylab("tSNE 2")
```

```
FeaturePlot(control.data, features = "Cxcl10") + theme(axis.text.x = element_blank(), axis.text.y = element_blank(),
    axis.ticks = element_blank()) + xlab("tSNE 1") + ylab("tSNE 2")
```

```
# Seurat v4
top10 <- control.data.all.markers %>%
    group_by(cluster) %>%
    top_n(n = 10, wt = avg_log2FC)

DoHeatmap(control.data, features = top10$gene, group.by = "seurat_clusters")
```

---

# Immune/Inflammatory Gene Set (Pdgfra and Gpr17 removed)

```
inflammation_gene_list <- list(c("Hif1a", "Cxcl5", "Hmgb1", "Il17d", "Il7", "Tnfaip6", "Traf6",
    "Acp6", "Adam9", "Agtr1a", "Ampd3", "Ankrd17", "Arhgap35", "Atm", "Bves", "C2cd4a", "Calcrl",
    "Cd1d2", "Cd3d", "Chd2", "Chd7", "Chrna4", "Cited1", "Clock", "Cnot4", "Cr2", "Cyld", "Dennd1b",
    "Dock10", "Duoxa2", "Eif2ak1", "Ercc1", "Ercc2", "Etv4", "Fbxo7", "Flt3l", "Gdnf", "Gpr171",
    "Itgav", "Klre1", "Ldlr", "Lrp1", "Lrtm2", "Masp2", "Med23", "Ncor1", "Nhej1", "Nkx2-1", "Oas3",
    "Orai1", "Pde4b", "Pou1f1", "Pou4f1", "Prkcq", "Prrc2c", "Ptpre", "Ptprz1", "Raf1", "Rgcc",
    "Rrs1", "Rtkn2", "Ryk", "Sema3d", "Sox6", "Src", "Srp54b", "Susd4", "Tet2", "Tmem176b", "Trim11",
    "Trim62", "Trpv4", "Tspan6", "Ufl1", "Unc5c", "Vav3", "Zcchc3", "Itgb8", "Ddx17", "Ep300", "Ccdc134",
    "Ifitm7", "Masp1", "Il1rap", "Sema5b", "Dscam", "Dll1", "H2-T3", "Tnfsf9", "Dcc", "Socs6", "Rbm14",
    "Ppp1r14b", "Zfp950"))
control.data <- AddModuleScore(object = control.data, features = inflammation_gene_list, name = "INFL")

FeaturePlot(object = control.data, features = "INFL1") + theme(axis.text.x = element_blank(), axis.text.y = element_blank(),
    axis.ticks = element_blank()) + ggtitle("Immune/Inflammatory") + xlab("tSNE 1") + ylab("tSNE 2")
```

```
VlnPlot(control.data, features = "INFL1") + ggtitle("Immune/Inflammatory") + xlab("Cluster") + ylab("Module Expression Score") +
    ylim(-0.1, 0.45) + NoLegend()
```
